# Rac2 Controls Tumor Growth, Metastasis and M1-M2 Macrophage Differentiation *In Vivo*

**DOI:** 10.1371/journal.pone.0095893

**Published:** 2014-04-25

**Authors:** Shweta Joshi, Alok R. Singh, Muamera Zulcic, Lei Bao, Karen Messer, Trey Ideker, Janusz Dutkowski, Donald L. Durden

**Affiliations:** 1 UCSD Department of Pediatrics, Moores Cancer Center, University of California San Diego, La Jolla, California, United States of America; 2 UCSD Department of Biostatistics, Moores Cancer Center, University of California San Diego, La Jolla, California, United States of America; 3 Department of Medicine, University of California San Diego, La Jolla, California, United States of America; 4 Department of Pediatrics and Rady Children's Hospital, San Diego, La Jolla, California, United States of America; BloodCenter of Wisconsin, United States of America

## Abstract

Although it is well-established that the macrophage M1 to M2 transition plays a role in tumor progression, the molecular basis for this process remains incompletely understood. Herein, we demonstrate that the small GTPase, Rac2 controls macrophage M1 to M2 differentiation and the metastatic phenotype *in vivo*. Using a genetic approach, combined with syngeneic and orthotopic tumor models we demonstrate that Rac2-/- mice display a marked defect in tumor growth, angiogenesis and metastasis. Microarray, RT-PCR and metabolomic analysis on bone marrow derived macrophages isolated from the Rac2-/- mice identify an important role for Rac2 in M2 macrophage differentiation. Furthermore, we define a novel molecular mechanism by which signals transmitted from the extracellular matrix via the α_4_β_1_ integrin and MCSF receptor lead to the activation of Rac2 and potentially regulate macrophage M2 differentiation. Collectively, our findings demonstrate a macrophage autonomous process by which the Rac2 GTPase is activated downstream of the α_4_β_1_ integrin and the MCSF receptor to control tumor growth, metastasis and macrophage differentiation into the M2 phenotype. Finally, using gene expression and metabolomic data from our Rac2-/- model, and information related to M1-M2 macrophage differentiation curated from the literature we executed a systems biologic analysis of hierarchical protein-protein interaction networks in an effort to develop an iterative interactome map which will predict additional mechanisms by which Rac2 may coordinately control macrophage M1 to M2 differentiation and metastasis.

## Introduction

Rac2 is a well-studied small GTPase that is known to function in hematopoietic and endothelial cell integrin and immunoreceptor signaling [1], [2]. Rac2 belongs to a family of 3 highly conserved Rac proteins, Rac 1, 2 and 3 [3], [4]. Rac2 is only expressed in hematopoietic and endothelial cells whereas Rac1 and Rac3 are ubiquitously expressed in mammalian systems [3], [4], [5]. Despite the high degree of sequence conservation among the three Rac isoforms, the Rac2 knockout mice display a number of hematopoietic defects mostly in the context of blood cell-specific receptor function or hematopoietic-specific effector mechanisms and also in kinase pathway-activated cell survival [1], [2], [6], [7], [8]. Rac2-deficiency has also been shown to impact B- and T-cell migration, activation, development (to a lesser extent in T-cells) [9], [10], [11], [12] and, in some reports, T-cell differentiation into T-helper type 1 (Th1) cells [9]. Recent reports also suggest the contribution of Rac2 to host defense responses *in vivo* [6], [10], [13]. Our laboratory reported that Rac2 is important in macrophage and endothelial cell migration on specific provisional matrix proteins like vitronectin or fibronectin via α_v_β_3_ or α_4_β_1_ integrins, respectively and mediates signaling downstream of these specific integrins. We also demonstrated as a control that Rac2 knockout Mθ and ECs are normal with regard to migration on intact triple helical collagen via α_2_β_1_ integrins [1], [2]. Interestingly, the angiogenic defect we reported in the Rac2 knockout mouse model [1] seems to reflect a specific defect in postnatal angiogenesis in that these mice have no developmental angiogenic/vasculogenic defect. These findings suggest an interesting **hypothesis**; that Rac2 has evolved in macrophages to represent a novel mechanism by which certain growth factors and the provisional integrins, α_4_β_1_ and α_v_β_3_, regulate the postnatal adaptive stromal angiogenic/wound healing response [14], [15], [16], [17].

Tumor inflammation has emerged as an important topic in cancer biology [18]. A defining feature of tumor inflammation is the polarization of M1 into M2 macrophages which promotes tumor growth, angiogenesis, invasion and metastasis. M1 macrophages are IL-12high, IL-23high, IL-10low; produce high levels of inducible nitric oxide synthetase (iNOS); secrete inflammatory cytokines such as IL-1β, IL-6, and TNF; and are inducer and effector cells in Th1 type inflammatory responses [19]. In contrast, M2 macrophages are involved in polarized Th2 inflammatory reactions and characterized by expression of arginase-1 and mannose and scavenger receptors [19], [20]. The separation of macrophages into populations of M1 and M2 subtypes is likely to represent a somewhat inexact and artificial classification, since macrophages display a high degree of heterogeneity and plasticity and a number of diverse phenotypes under different physiologic and pathologic conditions [21]. Therefore, investigators have suggested that Mθ differentiation is best represented as a bidirectional continuum and not by just two defined subgroups, M1 and M2 [22].

Although recent landmark reviews throw light on the signaling pathways connecting inflammation to cancer, the mechanism(s) that control the M1-M2 transition remain to be determined [23], [24], [25]. Moreover, many questions remain as to how signals are transmitted from the extracellular milieu to regulate the M1 to M2 transition, and how different myeloid cells exert control over tumor growth, invasion and metastasis. In this report, we have discovered a novel signaling pathway within the tumor microenvironment (TME) downstream of the MCSF receptor, the extracellular matrix and the α_4_β_1_ integrin in which Rac2 is necessary and sufficient to control, in a macrophage autonomous manner, tumor progression including the processes of tumor growth, invasion, angiogenesis, metastasis and polarization of M2 macrophages.

## Results

### Rac2 promotes tumor growth, angiogenesis and invasion

**Figure 1:**
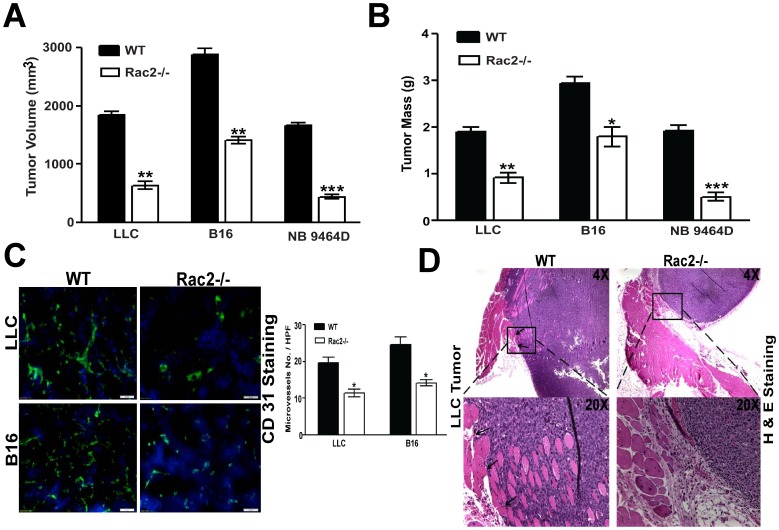
Rac2 promotes tumor growth, angiogenesis and invasion. (A & B) WT and Rac2-/- mice (n = 8-10) were subcutaneously implanted with 1×10^5^ LLC, B16F10 and 2×10^6^ NB9464D cells on the dorsal flank. Tumor growth was monitored regularly, until tumors were harvested on day 21. Mean tumor volume (A) and mass (B) for each group (n = 8) is plotted. Graphs present mean ± SEM of 8 mice in each group. Statistical significance is assessed by two sample *t*-test where *denotes *P*<0.05, ** denotes *P*<0.01 and *** denotes *P*<0.001. Experiment was repeated 7–8 times with similar results. (C) Left panel shows representative immunofluorescent staining of tumor vasculature by CD31 (green) and counterstain by DAPI (blue) on frozen tumor sections of LLC and B16 tumors implanted subcutaneously in WT and Rac2-/- mice. Right panel shows reduced microvascular density (MVD) in tumors isolated from Rac2-/- animals as compared to WT animals. MVD was determined by counting the number of microvessels per high-power field (HPF) in the section with an antibody reactive to CD31. Microvessels were counted blindly in 5–10 randomly chosen fields and data is representative of three independent experiments with 4–5 mice. * *P*<0.05 vs. WT. (D) H & E stained images (magnification 4X and 20X) showing invasive interface between the skin and muscularis layer of subcutaneous implanted LLC tumor into WT and Rac2-/- animals. The invasive interface is shown by arrows. Same results were obtained with 7–8 mice in each group and experiment is repeated 7–8 times.

As tumor growth, angiogenesis and invasion are prerequisites to tumor progression and metastasis, we first examined the effects of homozygous Rac2 deletion on these three phenotypes. Our initial observation was that tumor growth of 4 different syngeneic tumors which included: Lewis lung carcinoma (LLC), B16 melanoma and neuroblastoma (9464D) was significantly reduced in the Rac2-/- mice. (Figure 1A–B p<0.001). In a previous report, we observed a postnatal neovascularization defect in Rac2-/- mice [1]. This prompted us to determine if the reduced tumor growth observed in the Rac2-/- mice reflected an alteration in tumor-induced angiogenesis. Interestingly, our immunofluoresence analysis of microvessel density using CD31 staining showed that angiogenesis was greatly reduced in subcutaneously implanted LLC and B16 tumors in the Rac2-/- mice compared with WT animals (P<0.05; Figure 1C). More recently, we and other laboratories have confirmed that these Rac2-/- mice do not exhibit any defects in organ development. In this regard, we have performed an extensive set of experiments to evaluate whether Rac2 loss would disrupt the regulation of normal embryonic angiogenesis using experiments designed to evaluate the delicate processes that regulate the neovascular response within the neonatal retina (Figure S1A). From our results, we conclude that Rac2 is not required for angiogenesis during normal development but is required for the postnatal adaptive angiogenic responses involving wound healing [1] and/or tumorigenesis. These data clearly establish a role for Rac2 in tumor growth and postnatal tumor-induced angiogenesis. During the course of our tumor growth experiments we noted that tumor growth in the Rac2-/- mice appeared more encapsulated as compared to the invasive properties of subcutaneous tumor growth in the wild type mice, an observation which led to us to question whether Rac2 deletion may have an effect on the local invasive properties of tumor *in vivo*. We designed experiments to evaluate the invasive edge of subcutaneously implanted tumor cells into and through the muscularis layer of the body wall. H & E staining clearly shows a marked increase in tumor invasion within the muscularis layer in WT animals as compared to Rac2-/- animals injected with LLC tumors (Figure 1D). Taken together, these results suggest an important role for Rac2 in tumor growth, angiogenesis and invasion.

### Rac2 promotes tumor metastasis

**Figure 2:**
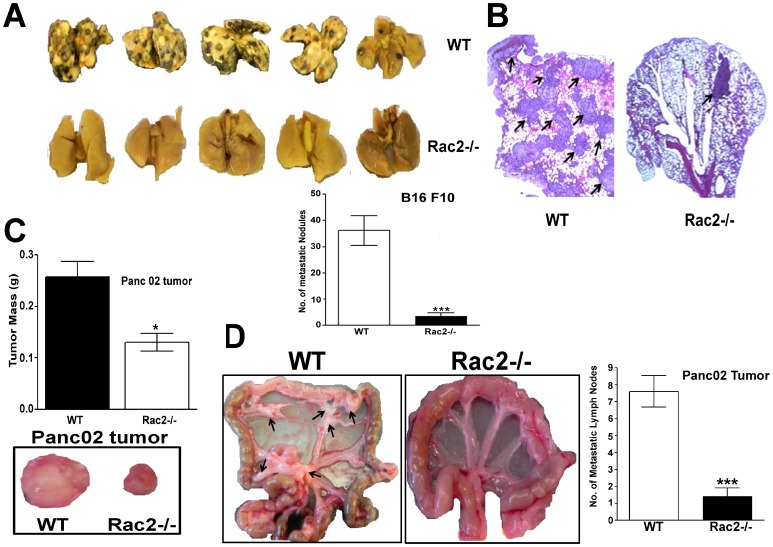
Rac2 promotes both experimental as well as spontaneous metastasis. (A) Experimental metastasis of B16 melanoma cells in WT and Rac2-/- mice (n = 5). B16 F10 melanoma cells (5×10^5^ cells) were injected through the tail vein, and after 15 days, the lungs were removed and the photographs were taken shown as upper panel in the figure. Lower panel shows mean number of tumor nodules visible on the surface of the lungs in WT and Rac2-/- mice. Surface tumor nodules in lungs were counted under dissecting microscope. Values are mean ± SEM (*n* = 5 or 6; *P*<0.001; pair wise two-sided Student's *t* test). A marked suppression in the number of metastatic nodules was observed in Rac2-/- mice.(B) H&E-stained lung tissues demonstrating large macroscopic nodules (black arrows) were greatly increased in number and size in the WT vs. Rac2-/- mice, 15 d after B16 tumor cell injection. (C) Upper panel shows tumor mass of pancreatic tumors implanted orthotopically in WT and Rac2-/- mice. Panc02 (1×10^6^) cells were injected in the pancreas of WT and Rac2-/- mice (n = 10). Tumors were removed 30 days after tumor implantation. Values are mean ± SEM (*n* = 10; P<0.05; pair wise two-sided Student's *t* test). Bottom panel shows representative images of pancreatic tumors isolated from pancreas of WT and Rac2-/- mice. (D) Left panel shows macroscopic view of Panc02 metastatic mesenteric lymph nodes from WT and Rac2-/- mice. Right panel shows number of metastatic mesenteric lymph nodes/mesentery. Values are mean ± SEM (*n* = 5 or 6; P<0.001; pair wise two-sided Student's *t* test). The data are representative of three independent experiments performed.

Tumor invasion is a preliminary step for metastasis; hence we used both experimental as well as spontaneous metastasis mouse models to examine metastasis in the Rac2-/- mice. B16F10 is considered a reliable method to study experimental metastasis in the C57BL background [26]. We observed a marked increase in metastatic foci in the lungs of WT mice compared with an 80% reduction in the number of nodules in Rac2-/- mice injected with B16F10 melanoma (p<0.001; Figure 2A–B). Moreover, we observed a significant reduction of tumor growth in orthotopic pancreatic Panc02 carcinoma, a model used to study spontaneous lymph node metastasis (P<0.05; Figure 2C–D). Importantly, Rac2-/- mice orthotopically implanted with Panc02 cells in pancreas display a marked reduction in regional colonic lymph node metastasis (Figure 2D & Figure S2) suggesting an important role for Rac2 in controlling spontaneous lymph node metastasis.

### Role of Rac2 in macrophage migration, extravaseation and α_4_β_1_/CSF1 receptor signaling

**Figure 3:**
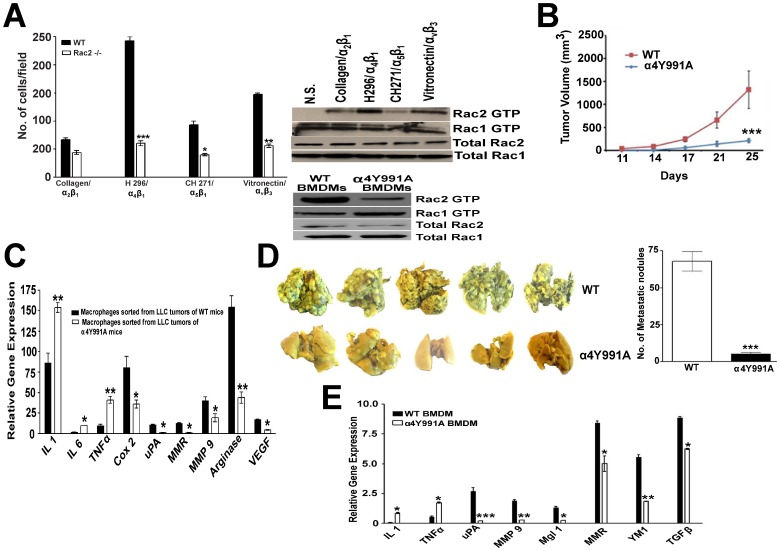
Rac2 signaling is required for specific α_4_β_1_ integrin signaling. (A) Left panel shows BMDMs from WT, and Rac2-/- mice were tested in haptotaxis assay for capacity to migrate on different matrix proteins or fragments of fibronectin, vitronectin (via α_v_β_3_/α_v_β_5_); H296, via α_4_β_1_; CH271, via α_5_β_1_ and collagen via α_2_β_1_/α_2_β_2_. Comparison of WT to Rac2-/- BMDMs shows significant difference on H296 peptide (*P*<0.001), CH271 peptide (*P*<0.05), VN protein (*P*<0.01). Data represent mean ± SEM, representative of 4 independent experiments performed (n = 3). Right Upper panel shows Rac2 pull down assay indicating the extent of Rac2 activation in WT BMDMs under conditions of adhesion to: NS, no stimulation; Vitronectin (α_v_β_3_/α_v_β_5_); H296, fibronectin fragment for α_4_β_1_; CH271, fibronectin fragment for α_5_β_1_; and Collagen (α_2_β_1_/α_2_β_2_). Conversion of GDP-Rac2/Rac1 to GTP-Rac2/Rac1 was determined by using GST fusion protein representing the GTP-Rac-binding CRIB domain of the PAK-1 kinase. Cell lysate used in this comparison contained equal amounts of protein per lane. Total Rac1 and Rac2 protein was loaded for control. Right Lower panel shows Rac2 pull down assay indicating the extent of Rac2 activation in WT and α4Y991A knock in BMDMs under conditions of adhesion to H296, fibronectin fragment for α_4_β_1._ Experiments were repeated 2-3 times with similar results. (B) LLC cells were inoculated subcutaneously in WT and α4Y991A mice (n = 6–8) and tumor growth was recorded as described in Methods. Values represent mean ± SEM (*n* = 6–8 mice per group; P<0.001) (C) Quantitative PCR analysis of mRNA for M1, M2 specific genes in the macrophages sorted from LLC tumors grown in WT and α4Y991A mice (n = 3–4). LLC tumors implanted in WT and α4Y991A mice were used for FACS sorting of macrophages on the basis of F4/80 and CD11b staining as described in Materials and Methods. RNA was isolated from these macrophages and was used for real-time PCR analysis of the indicated genes described in Methods. Values are mean ± SEM (*n* = 3–4). Statistical significance is assessed by two sample *t*-test where *denotes *P*<0.05, ** denotes *P*<0.01 and *** denotes *P*<0.001.). (D) Left panel shows representative photograph of pulmonary metastatic foci produced 15 days after intravenous injection of B16F10 cells in WT and α4Y991A mice (n = 6–8). Right panel shows mean number of tumor nodules visible on the surface of the lungs in WT and α4Y991A mice. Values are mean ± SEM (n = 6; P<0.001; pair wise two-sided Student's *t* test). (E), Quantitative PCR analysis of mRNA for IL 1, uPA, TNFα, MMP9, Mgl1, MMR, YM1 and TGF-β in BMDMs isolated from WT and α4Y991A knock in mice and cultured in MCSF *in vitro*. Data are representative of three independent experiments, shown are mean ± SEM, *P<0.05, **P<0.01 and ***P<0.001 vs. WT, t test.

Our results clearly establish a requirement for Rac2 in tumor growth, invasion and metastasis. The question which now strikes out to be answered is how this small GTPase, which is specifically expressed in the hematopoietic compartment, modulates tumor growth, metastasis and the M1 to M2 transition in macrophages? A growing body of evidence suggests that Mθs are frequently found to infiltrate tumors and have been linked to diverse tumor-promoting activities [27], [28]. Hence, we investigated if there is any defect in the recruitment of macrophages in the tumor in these Rac2-/- mice or if this GTPase modulates the phenotype of macrophages recruited into the tumor via some other mechanism? We reasoned that specific cell surface receptors e.g. certain integrins and/or growth factor receptors in the TME (e.g. MCSF receptor) could signal through Rac2 to orchestrate macrophage differentiation and promote metastasis *in vivo*. Previous work from our laboratory demonstrated that specific α_4_β_1_ and α_v_β_3_/α_v_β_5_ integrin directed migration in macrophages requires Syk and Rac2 [2]. In order to gain insight into the specificity of this signaling axis, we examined the extent to which integrin-specific engagement in macrophages leads to migration and the activation of Rac2 by quantitating Rac2-GTP levels following adhesion of WT macrophages to different extracellular matrices. The most dramatic effect is observed with the H296 a ligand for α_4_β_1_ followed by VN ligand for α_v_β_3_ (Figure 3A, left panel). Importantly, the defect in Rac2 activation directly correlates with quantitative data related to the effects of Rac2 deficiency on α_4_β_1_ vs. α_5_β_1_ dependent macrophage migration. In contrast, engagement of α_5_β_1_ with CH271 and α_2_β_1_ with collagen does not result in an appreciable activation of Rac2 and there is minimal migration defect on these matrix proteins observed in Rac2-/- murine macrophages (Figure 3A, right upper panel). We next examined if α4Y991A knock-in mice bearing a point mutation in α4 integrin tail (Y991A), has any defect in activating Rac2. Interestingly, Rac2 pull down experiments showed less Rac2-GTP activation under conditions of CSF1R/α_4_β_1_ engagement in α4Y991A knock-in mice as compared to WT mice (Figure 3A, right lower panel). This observation leads us to study the role of α_4_β_1_ specific integrin in tumor growth and M2 macrophage differentiation. We observed that the α4Y991A k/in mice are defective in tumor growth (p<0.001), polarization of macrophages and metastasis (by 90%, p<0.001) (Figure 3B–D). Consistent with a previous published report [29], parallel results from our laboratory confirm that macrophage entry into LLC tumors grown in α4Y991A knock-in mice is markedly reduced (50% reduction in F4/80 cells) (data not shown). To determine if the α4Y991A knock-in mice display a defect in M2 macrophage differentiation *in vitro* we utilized RT-PCR methods to quantitate the expression of M1 and M2 markers in the BMDMs isolated from α4Y991A knock-in animals and found that macrophages cultivated in MCSF, a ligand for the CSF1R and known to induce M2 differentiation, were defective in the process of M2 macrophage differentiation (Figure 3E). These results are consistent with our experimental model which predicts that an extracellular matrix interaction with the α_4_β_1_ integrin co-transmits a specific signal in concert with the CSF1R in macrophages via Rac2 to promote the M2 polarization of macrophages and tumor progression. In addition, from our data in Rac2-/- mice, we conclude that macrophage entry into the tumor is likely **necessary but** **not sufficient** to drive M1 to M2 differentiation of TAMs.

### Rac2 promotes the differentiation of macrophages into M2 phenotype *in vitro and in vivo*

**Figure 4:**
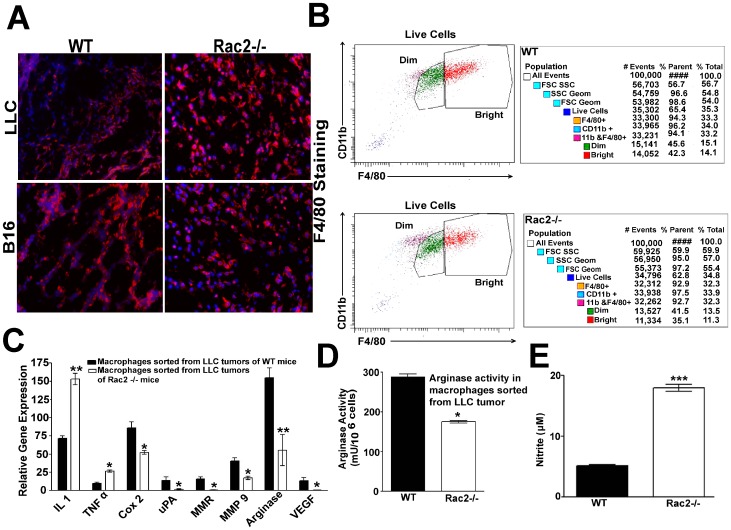
Rac2 promotes M2 macrophage polarization with no substantial change in macrophage recruitment. (A) Identification of F4/80^+^ macrophages by immunofluorescence microscopy in the frozen sections of LLC and B16 tumors stained with antibodies against F4/80 and imaged by fluorescence microscopy. The average no. of macrophages per HPF for 3 different experiments were 42±8, (WT) 38±6 (Rac2-/-) for LLC tumors and 50±5, (WT) 45±10, (Rac2-/-) for B16 tumors. Macrophages were counted blindly by 3 individuals in 5-10 randomly chosen fields and data is representative of three independent experiments with 4 mice. (B) Figure represents FACS data showing the quantification of CD11b and F480^+^ macrophages infiltrated in LLC tumors implanted in WT and Rac2-/- mice. Experiment was repeated 4-5 times with 3-4 mice in each group and similar results were obtained. (C) Quantitative PCR analysis of mRNA for M1, M2 specific genes in the macrophages sorted from LLC tumors grown in WT and Rac2-/- mice (n = 3–4) as described in Methods. Values are mean ± SEM. Statistical significance is assessed by two sample *t*-test where *denotes *P*<0.05, ** denotes *P*<0.01 and *** denotes *P*<0.001. (D) Arginase activity was measured in macrophages sorted from LLC tumors injected in WT and Rac2-/- mice as described in Methods. (E) Nitrite production in macrophages sorted from LLC tumors injected in WT and Rac2-/- and stimulated with 10 ng/ml LPS for 24 h. Supernatants were collected, and nitrite concentration was measured as described in Methods. Results are mean ± SEM (n = 3–4 mice) for 3 independent experiments performed in triplicate (P<0.05 for arginase activity and *P*<0.001 for nitrite assay; student's t test).

The above results establish a role for the Rac2 in macrophage migration via the α_4_β_1_ integrin. These observations raise a number of important questions; how does Rac2 exert its regulatory effects on macrophage differentiation and metastasis? Since macrophage entry into the tumor microenvironment (TME) in the Rac2-/-mice is normal, as revealed by F4/80 quantification and FACS analysis (Figure 4 A–B), the data suggest an alternative mechanism for the tumor growth and metastatic defect observed in the Rac2-/- mouse. Biswas et al has reported that the phenotype of tumor associated Mθs varies with the stage of tumor development [30]. In order to characterize Mθs present in tumors, Mθs were sorted from LLC tumors grown in WT and Rac2-/- animals, RNA extracted and used to determine the expression of M1 and M2 specific genes using real time PCR (RT-PCR). The expression levels of tumor promoting M2 markers: Cox 2, uPA, MMP9, MMR, arginase and VEGF are significantly higher in Mθs sorted from LLC tumors of WT while Mθs sorted from Rac2-/- mice displayed higher levels of proinflammatory cytokines, which are considered as M1 markers; IL1 and TNFα (Figure 4C). Moreover, higher arginase activity (p<0.05) and lower nitrite production (NOS) (p<0.001) was observed in macrophages isolated from LLC tumors injected into WT animals as compared to Rac2-/- mice (Figure 4D–E). These results further support our model that Rac2 is required to promote tumor growth, invasion, angiogenesis and metastasis potentially by promoting alternative activation of macrophages into the M2 phenotype.

**Figure 5:**
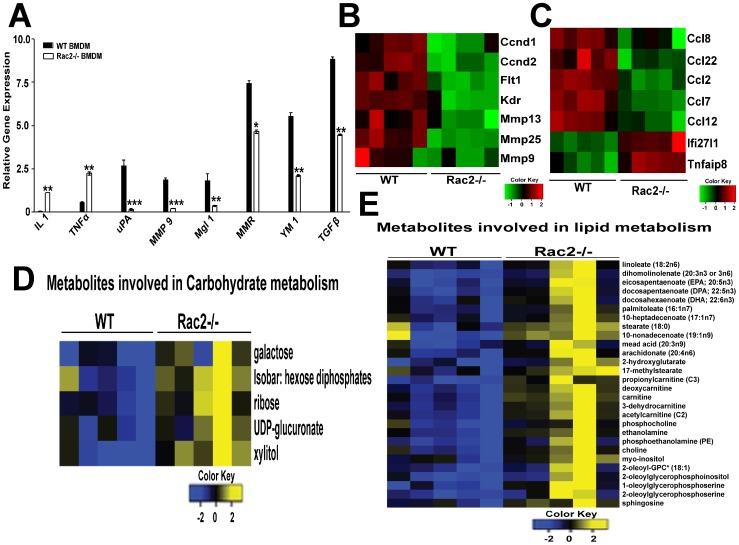
Rac2 promotes differentiation of M2 macrophages *in vitro*. (A) Quantitative PCR analysis of mRNA for IL 1, uPA, TNFα, MMP9, Mgl1, MMR, YM1 and TGF-β in BMDMs isolated from WT and Rac2-/- mice and cultured in MCSF *in vitro*. Values are mean ± SEM (n = 3-4 mice). Statistical significance is assessed by two sample *t*-test where *denotes *P*<0.05, ** denotes *P*<0.01 and *** denotes *P*<0.001. (B) & (C), Heat map generated from microarray analysis of BMDMs isolated from WT and Rac2-/- mice (n = 5 in each group) as described in Materials and Methods. Colors illustrate fold changes, Red: up-regulation; green: down-regulation; black: no change. The bar code on the bottom represents the color scale of the log 2 values. The differential expression of genes related to cell cycle, angiogenesis and invasion are shown in B and M1-M2 polarization are shown in (C). (D) & (E), Heatmap representation of metabolites across BMDMs from WT (n = 5) and Rac2-/- (n = 5) mice. Shades of yellow represent elevation of a metabolite and shades of blue represent decrease of a metabolite relative to the median metabolite levels (see color scale). Colors illustrate fold changes, Yellow: up-regulation; blue: down-regulation; black: no change. Data shows higher expression of metabolites related to carbohydrate (D) and lipid metabolism (E) in Rac2-/- BMDMs.

On the basis of these observations (Figure 1–4) and the importance of M2 macrophages in tumor progression [27], [28], we hypothesized that Rac2 is somehow regulating the transition of macrophages into the M2 phenotype *in vitro*. To further test this hypothesis, we used RT-PCR to detect the expression of M1 and M2 markers in the BMDMs isolated from WT and Rac2-/-, animals. We found that Rac2-/- mice were defective in the differentiation of macrophages into M2 phenotype *in vitro* (Figure 5A). Similar to BMDMs, the peritoneal macrophages isolated from Rac2-/- mice showed marked defect in arginase activity (data not shown). These interesting observations prompted us to conduct mRNA gene expression and metabolomic studies on Rac2-/- vs. WT BMDMs to identify other components downstream of Rac2 that might be required for tumor growth, metastasis and polarization of macrophages (Figure 5 B–E and Figure S3).

### Genomic studies on Mθs

For microarray data analysis, we conducted GSEA analysis [31] using the KEGG (Kyoto Encyclopedia of Genes and Genomes) database [32]. The analysis confirms that BMDMs isolated from WT mice express Rac2 as compared to negative result in Rac2-/- BMDMs. Interestingly, the expression of genes related to cell cycle, invasion and angiogenesis are significantly enriched in WT BMDMs (Figure 5B & Table S1). These results are consistent with the high levels of the angiogenic cytokine, VEGF and matrix degrading enzyme, MMP9 observed in our RT-PCR analysis of tumor derived Mθs in WT vs. Rac2-/- mice (Figure 4C). Other genes differentially expressed in WT vs. Rac2-/- BMDMs included prototypic M2 markers; CCL2, CCL22 [33], and genes with high expression in Rac2-/- BMDMs included prototypic M1 markers; IFNα inducible protein (Ifi27l1) and TNFα inducible protein (Tnfaip8) [19] (Figure 5C). Recent transcriptional profiling comparing human monocytes to the macrophage **lineage and** Mθ polarization has revealed new genes which are differentially expressed in M1 vs. M2 macrophages [34]. Interestingly, we also observed significant high expression of apoptosis related protein (Bnip3), extracellular mediator (IGFBP4), enzymes related to carbohydrate metabolism (Pfkl, Aldoc, Ldhb, Pgk1) and nucleotide metabolism (Uck2, Impdh, Ak3l1) in Rac2-/- BMDMs, which are reported to be high in M1 macrophages [34]. In the same context, we observed increased expression of membrane receptors (Ms4a4c, Ms4a4d, Hrh2) and extracellular mediator (Fgl2) in WT BMDMs which are reported to be expressed in M2 macrophages [34] (Figure S3A). Most notably, our microarray analysis data suggest significant expression of some chemokines (CCL8, 12), solute carrier proteins (Slc28a2, Slc4a7, Slc25a23, Slc38a1, Slc44a1, Sort1), G-protein coupled receptor (Gpr31c, Gpr128), membrane receptors (Tmem 209, Rell1, Ccr1l1, xcr1, Ahr, Insr), macrophage activation 2-like molecule, members of schlafen family (slfn1,4 and 9) which have not been classified in M1 and M2 paradigm but have important functions in macrophage activation and differentiation. Taken together, our microarray results identified additional target genes controlled by Rac2 which correlate *in vivo* with M1 or M2 differentiation and independently support RT-PCR results generated in our knockout models that WT and Rac2-/- BMDMs are M2 and M1 skewed, respectively.

### Metabolomic studies of Mθs

Recent metabolomic analysis done on macrophages suggest that classically activated M1 macrophages show relatively elevated glycolysis and oxidative pentose phosphate pathway (PPP) but reduced oxygen consumption via the TCA cycle compared to M2 cells [35]. The analysis of our metabolomic data suggest that the metabolites related to carbohydrate, lipid and nucleotide metabolism are higher in Rac2-/- BMDMs (Figure 5D-E and Figure S3 B). These results are consistent with our genomic data showing high-level expression of enzymes related to carbohydrate and nucleotide metabolism in Rac2-/- BMDMs (Figure S3A). We observed that Rac2-/- BMDMs have a higher rate of glucose utilization via glycolysis as revealed by decreased glucose levels but increased glucose 6-phosphate and fructose 6-phosphate and lactate levels. We observed evidence of augmented pentose phosphate shunt activity in Rac2-/- BMDMs, indicated by increase levels of ribose and xylitol biochemicals. This pathway is associated with presence of pentose alcohols which lead to augmented nucleotide metabolism (Figure 5D). Collectively, these genomic and metabolomic data serve to identify new biomarkers for M1 and M2 Mθdifferentiation and further support our hypothesis that Rac2 plays a unique role in transition of macrophages to anti-inflammatory M2 phenotype to promote tumor metastasis.

### MCSF receptor co-signals through α_4_β_1_ integrin to activate Rac2 to the GTP-bound state

Our results establish a role for the Rac2 GTPase downstream of α_4_β_1_ integrin to control macrophage migration [2] (Figure 3A). These observations lead us to investigate if stimulus from CSF-1 and/or α_4_β_1_ integrin is sufficient to promote Rac2 activation and M2 macrophage differentiation. Considerable evidence exists to support the fact that growth factor receptors like the CSF-1 receptor (FMS) co-signal through integrins [36]. Lawrence et al has suggested that MCSF receptor activates IRF4 transcription factor to promote M2 differentiation of macrophages [37]. In addition, clinical studies in cancer implicate the MCSF signaling network as a negative prognostic component in breast cancer [38]. To gain insight into the potential role of the CSF1 and α_4_β_1_ receptors in Rac2 activation and macrophage M2 differentiation, we took advantage of the reports that M2 macrophages can be generated under conditions of MCSF stimulation *in vitro* [39], [40]. Consistent with this, real time PCR analysis done on macrophages cultured in MCSF (BMDM) or GMCSF (GBMDM) demonstrated that GBMDM express higher levels of proinflammatory cytokines, while BMDM express increased levels of Mgl 1 and MMR mRNA which are considered as M2 markers (Figure S4A). In support of our results, literature suggests that on the basis of respective cytokine profiles, macrophages generated in the presence of GMCSF or MCSF [40] display differences in cytokine expression and are considered proinflammatory or anti-inflammatory macrophages, M1 vs. M2 respectively [40]. Importantly, macrophages cultured in MCSF (BMDM) and GMCSF (GBMDM) are found to be ≈90% pure on the basis of F4/80 and CD11b staining by FACS (data not shown).

**Figure 6:**
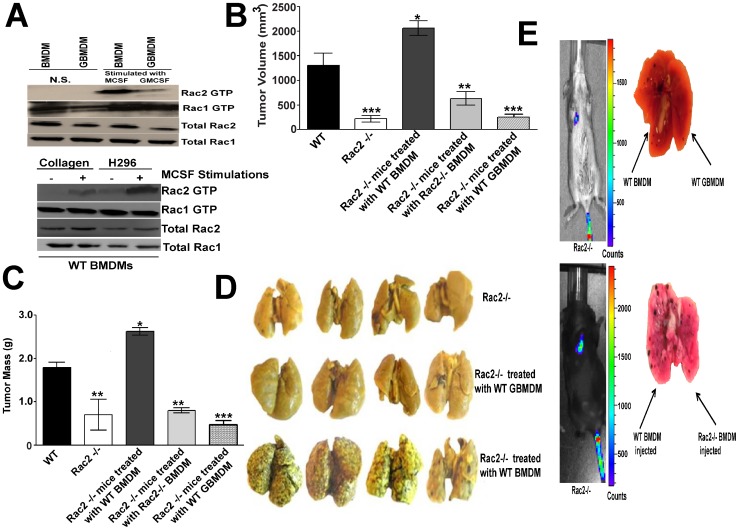
Reversal of the metastatic defect by injection of macrophages cultured in MCSF. (A) Upper panel shows that MCSF signaling differentially activates Rac2 while GMCSF signaling does not result in significant Rac2 activation. BMDMs or GBMDMs cultured in MCSF or GMCSF for 7 days were serum starved for 4 hrs and stimulated with 50 ng/ml of MCSF or GMCSF for 15 min followed by Rac2 GTP pull down assays as described in Materials and Methods. Lower panel shows the differential activation of Rac2 when Mθs are costimulated through the MCSFR and α_4_β_1_ integrin. WT BMDMs cultured in MCSF for 7 days were serum starved for 4 hrs, trypsinized and were allowed to engage with α_4_β_1_ ligand (H296) or α_2_β_1_ ligand (collagen), followed by MCSF stimulation (50 ng/ml) for 15 min and Rac2 GTP pull down assay as described before. (B & C) Tumor volume (B) and mass (C) of LLC tumors grown in Rac2-/- mice treated with or without 1 million WT BMDMs or Rac2-/- BMDMs or WT GBMDMs. After 5 days of LLC tumor inoculation, Rac2-/- mice were treated either with 1 million WT BMDMs or GBMDMs or Rac2-/- BMDMs or every third day, until tumors were removed on day 21. Values are mean ± SEM (*n* = 6-8). Statistical significance is assessed by two sample *t*-test where *denotes *P*<0.05, ** denotes *P*<0.01 and *** denotes *P*<0.001. Experiment was repeated three times with similar results. (D) Reversal of B16 metastasis by injection of WT BMDMs and not by WT GBMDMs in Rac2-/- mice. Figure shows representative photograph of pulmonary metastatic foci produced 15 days after intravenous injection of B16F10 cells in Rac2-/- mice and treated with 1 million WT BMDMs or WT GBMDMs. One dose of 1 million BMDMs or GBMDMs were given to Rac2-/- mice two days before inoculating 5×10^5^ B16F10 cells intravenously followed by treatment with 1 million BMDM or GBMDM every third day, lungs were harvested on day 15. Data are representative of three independent experiments with 6–8 mice in each group. (E) Reversal of metastatic defect in Rac2-/- mice by local injections of 1 million WT BMDMs in the right lobe of lungs and 1 million WT GBMDMs or Rac2-/- BMDMs in the left lobe of Rac2-/- mice, followed by tail vein injections of B16 luciferase cells (5×10^5^) after 2 days. The luciferase signal was monitored every third day on IVIS by injecting luciferin, until lungs were harvested on day 15 (n = 5). Data are representative of two independent experiments with 5–6 mice in each group.

We hypothesize that if CSF-1 and/or α_4_β_1_ receptor engagement drives M2 differentiation via the Rac2 axis, we would expect that MCSF and/or α_4_β_1_ stimulation would preferentially activate Rac2 (and not Rac1) to its GTP bound state in Mθs bound to H296, a ligand for α_4_β_1_. In order to gain insight into the specificity that MCSF activates Rac2, we examined the effect of MCSF vs. GMCSF stimulation on the activation of Rac2 vs. Rac1 in macrophages. As anticipated, MCSF stimulation of MCSF cultivated Mθs differentially activate Rac2 and not Rac1 to the GTP-bound state (5-fold increase Rac2 vs. Rac1) (Upper panel, Figure 6A). In order to determine if α_4_β_1_ activates Rac2, we examined the effect of macrophage stimulation via different extracellular matrix stimulation on the activation of Rac2 vs. Rac1. For this, WT macrophages plated on H296, a ligand for α_4_β_1_ or on collagen, a ligand for α_2_β_1_, were stimulated with or without MCSF. Our results clearly provide evidence that higher levels of Rac2-GTP is activated when cells are engaged with α_4_β_1_ ligand and stimulated by MCSF (Lower panel, Figure 6A). Taken together, we conclude that MCSF co-signals with α_4_β_1_ integrin to activate Rac2. Our current results establish that the Rac2 GTPase controls tumor growth, invasion and metastasis and that Rac2 is activated downstream of the α_4_β_1_integrin, the CSF1 receptor and that Rac2 is required for macrophage differentiation. We continue to actively investigate the critical components of Rac2 signaling which are necessary and sufficient to drive macrophage M2 transition and important phenotypes like metastasis.

### Macrophage autonomous nature of Rac2 defect in the promotion of tumor growth, metastasis and M1-M2 transition

Since Rac2 is expressed in a number of hematopoietic lineages and in endothelial cells [5], we sought to determine if the tumor growth, metastasis and M1-M2 specific defects noted in the Rac2-/- mouse model were macrophage autonomous. If so, we would predict that the injection of WT BMDMs and not BMDMs isolated from Rac2-/- mice into Rac2-/- mice would reverse the metastasis and M1-M2 defects *in vivo*. In addition, BMDMs from WT mice (M2) and not GBMDMs (M1) upon injection into Rac2-/- mice would reverse the metastatic phenotype *in vivo*. As predicted, the injection of WT BMDMs and not WT GBMDMs or Rac2-/- BMDMs increased tumor growth, metastasis and polarization of macrophages to M2 phenotype in Rac2-/- animals (Figure 6 B-D and Figure S4 B-C). These three independent experimental observations and datasets support the hypothesis that reversion of tumor growth and metastasis in Rac2-/- mice is a **macrophage autonomous phenotype**. The above experiments were done with 5-6 daily injections of 1×10^6^ macrophages through tail vein, followed by tail vein challenge with 5×10^5^ B16F10 melanoma cells on day 5. Under these conditions, WT and not Rac2-/- Mθ injections lead to reversal of tumor growth defect in Rac2-/- mice *in vivo* (Figure 6 B-C). To further support the role for Rac2 and macrophage autonomy in the control of metastasis, we performed simultaneous local injections of WT BMDMs into right hemithorax of Rac2-/- vs. injection of WT GBMDMs into the left hemithorax of the same Rac2-/- mouse, followed by B16F10 melanoma tail vein injections two days later. We then used luciferase tranfected B16F10 cells to image the signal coming from the metastatic tumor cells within the lung parenchyma. The right hemithorax (injected with WT BMDMs) showed luciferase activity and B16 metastatic nodules while there was no B16 melanoma or luciferase signal and minimal metastatic nodules detected within the left hemithorax (injected with WT GBMDM vs. Rac2-/- BMDM) (Figure 6E). These results were confirmed by H & E staining of B16F10 metastatic nodules (Figure S4D).

#### Molecular model for macrophage Rac2 signaling, M2 transition and metastasis; construction of a Rac2-M1-M2 macrophage protein-protein interactome map

**Figure 7:**
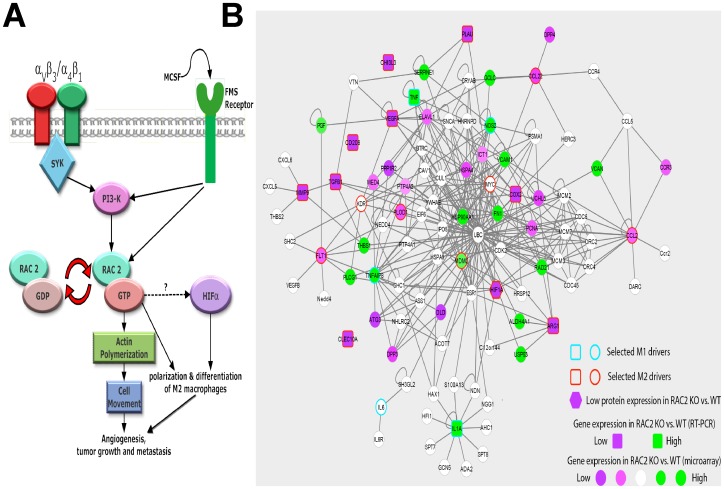
Identification of Rac2 regulated signaling pathways controlling differentiation of macrophages into M2 phenotype. (A) Graphic representation of novel integrin-Rac2 signaling axis in macrophages required for tumor growth, invasion, metastasis and the polarization of macrophages into M2 phenotype. (B) Interactome map developed from multiple-omic data which predicts how Rac2 regulates macrophage M2 differentiation. Nodes indicate genes either preselected for hierarchical analysis (colored borderer) or prioritized directly by the network propagation algorithm. Edges indicate protein-protein interactions. Node color indicates the protein or gene expression change in the Rac2-/- vs. WT condition.

Figure 7A shows a schematic representation of the signaling pathway elucidated in this report. The pathway extends from the cell surface receptors MCSF receptor and integrin α_4_β_1_ to the activation of the Rac2 to control differentiation of M2 macrophage differentiation, tumor growth and metastasis *in vivo*. Furthermore, we applied integrative network analysis to integrate literature curated results with our primary data (gene expression & metabolomic studies), and prioritize other candidate genes in close network neighborhood (see Methods). To this end we superimposed the M1 and M2 driver genes (previously established or validated in the Rac2-/- mice) upon a global map of protein-protein interactions. We then applied network propagation to identify a sub network of top 100 genes (listed as Table S2), including the known and candidate drivers of the Rac2-controlled macrophage M1 to M2 transition and metastasis (Figure 7B).

## Discussion

It is well-appreciated that alterations in the extracellular matrix (ECM) contribute to important biological events which include wound healing, inflammation, tumor progression and metastasis [41], [42], [43]. The current investigation began with our initial observation that the provisional integrins, α_4_β_1_/α_v_β_3_ induced migration requires a specific isoform of Rac, Rac2 in macrophages and endothelial cells and that this pathway regulated the postnatal angiogenic response *in vivo* [1], [2]. Integrin signaling and migration on type IV intact collagen via the α_2_β_1_ integrin was completely normal in the Rac2-/- mice (Figure 3). Importantly, in the Rac2-/- mice there is no evidence of a vascular defect suggesting this regulatory requirement existed only in postnatal period. This was an important distinction since in other knockout models e.g. VEGF-/- and +/- mice were embryonic lethal or associated with profound defects in vasculogenesis and developmental angiogenesis [44], [45]. Careful control *in vitro* experiments confirmed several important features of our Rac2-/- model: **1**) Rac2-/- macrophages are defective for migration on certain matrix proteins corresponding to specific integrins, in particular α_4_β_1_ and α_v_β_3_ 2) stable retroviral transfection of an epitope tagged Rac2 and not Rac1 into macrophages reversed the migration defect for these integrins and 3) minimal to no migration defect is seen for α_5_β_1_ or α_2_β_1_ in Rac2-/- Mθs, respectively [2] (Figure 3A). This lead to us to investigate an important question; how does a specific provisional integrin α_4_β_1_ in postnatal inflammatory pathophysiologic states transmit a signal to encode information about the ECM/TME to macrophages and endothelial cells to maintain control over inflammation and angiogenesis and how do these pathways interface with tumor promotion and tumor progression *in vivo*. Such a pathway would need to exquisitely turn on and turn off the processes of angiogenesis and inflammation in health and could be deregulated in disease. A discovery of the fundamental signaling mechanisms for the regulation of these ECM/TME driven processes could be of potential therapeutic importance.

The α_4_β_1_/α_v_β_3_ integrins are expressed in hematopoietic and endothelial cells [14] and play a key role in inflammation [15], [29], [46], [47]. Other investigators have shown that α_4_β_1_ is involved in tumor progression involving the TLR4 signaling pathway and p110γ axis. However, these studies fail to provide a comprehensive picture for how the α_4_β_1_ integrin signals to regulate M2 differentiation and metastasis [29]. As expected, our data demonstrate that the phenotype of the α4Y991A knock-in differs significantly from the Rac2 -/- mice in that α4Y991A knock-in mice as reported by Schmidt et al [29] display a defect in Mθ entry into the TME whereas no such defect is noted in Rac2-/- mice. We speculate that *in vivo* alternative mechanisms exist in Rac2-/- macrophages which allow TME infiltration to occur and hence the Rac2 defect *in vivo* is more significantly involved in Mθ M2 differentiation (Figure 4C). In future studies, we will utilize our Rac2-/- model and other knockout and knockin models to pick-apart the pathways required for the provisional integrins to control tumor growth and metastasis. Our present studies demonstrate that specific α_4_β_1_ integrin in macrophages regulates, tumor growth and metastasis (Figure 3) and that the mechanism for α_4_β_1_ induced migration and M2 macrophage differentiation requires Rac2.

In our previously published report, we utilized a reductionistic approach to map the signaling elements required for α_4_β_1_ to engage Rac2 and not Rac1 [2]. We transduced a nonmyeloid cell, COS7 which expresses Rac1 and α_4_β_1_ but not Rac2 or Syk kinase with Syk and/or Rac2 and were able to determine that Syk was necessary and sufficient to convert α_4_β_1_ integrin dependent migration in COS7 cells to Rac2 dependency [2]. To further support our model for how the provisional integrins are linked to tumor growth, metastasis and M2 macrophage differentiation, we provide evidence that MCSF receptor cosignals through α_4_β_1_ integrin to promote these events by specifically activating Rac2 (Figure 6A). Although there is no literature on the interaction of CSF-1 receptor and α_4_β_1_ integrin, a study by Faccio *et al* has illustrated that M-CSF stimulation induces association between CSF-1 receptor (FMS) and α_V_β_3_ integrin in osteoclasts [36]. Moreover, our results also suggest that only MCSF but not GMCSF stimulations activate Rac2 (Figure 6A). In support of our results, existing literature provides evidence that MCSF signaling activates its receptor the FMS tyrosine kinase which sequentially activates PI-3 kinase and Rac in microglia and bone marrow derived cells [48], [49].

Previous findings clearly demonstrate that in the absence of Rac2, macrophages and/or neutrophils display suppressed reactive oxygen species (ROS) production, defective chemotaxis, impaired phagocytosis, and decreased microbial killing [1], [2], [6], [7], [8]. In the present study, we provide a novel role of macrophage Rac2 in controlling tumor growth and M2 macrophage differentiation. The microarray studies conducted on BMDMs isolated from Rac2-/- mice provide evidence that Rac2 controls expression of genes related to invasion and angiogenesis which supports the RT PCR data obtained from tumor derived Mθs in WT vs Rac2-/- mice (Figure 4C). Moreover Rac2 also controls expression of CCL2 and CCL22 (Figure 4) which are considered as M2 markers [33]. Despite considerable progress, the molecular entities involved in the global rearrangement of the transcriptional profile occurring during alternative macrophage activation are still largely unknown. We will continue to examine our Rac2-/- model, which clearly establish a dominant role for Rac2 downstream of MCSF receptor and the α_4_β_1_ integrin in the control M2 macrophage differentiation. It is our view that the study of this model and other contributing elements (see interactome, Figure 7B) will further illuminate additional signaling pathways controlling this important tumor-specific phenotype.

We conclude that Rac2 provides the signaling specificity to drive the M2 macrophage phenotype under conditions of inflammation where macrophages are interacting with the provisional extracellular matrix and immunoregulation is key, e.g. parasitic infection. Importantly, malignant tumors have found a way to co-op this mechanism for M2 macrophage transition as an important component of tumor progression. The coordinate regulation of the macrophage transcriptomic program downstream of CSF1R/α_4_β_1_ stimulation is required for the complex physiologic transition from M1 to M2 remains unclear. We will continue to utilize our Rac2-/- model and our computational modeling methodologies [50], [51] to interatively implicate additional cooperating signaling elements i.e. protein-protein interactions in an effort to validate and then assemble a more complete picture of how the M1-M2 Mθ transition is regulated during the process of tumorigenesis.

## Methods

### Animal studies

All procedures involving animals were approved by the University of California San Diego Animal Care Committee, which serves to ensure that all federal guidelines concerning animal experimentation are met. Rac2-/- mice and normal littermates in C57BL/6J genetic background (backcrossed >50 generations into C57BL background) have been described [2]. Integrin α4Y991A mice were a gift from Dr. Mark Ginsberg [52].

### Antibodies and Reagents

Rac2 antibody is from Novus Biologicals. Rac1 antibody is obtained from Santa Cruz Biotechnology. Vitronectin and collagen are from Sigma (Sigma-Aldrich, St. Louis, MO), fragments of fibronectin (H296 and CH271) are from R&D systems. Primary or fluorescent antibodies against CD31 (clone MEC13.3), CD11b (clone M1/70) are from BD Biosciences, F4/80 (clone BM8) is from eBiosciences. 4, 6 diamidino-2-phenylindole (DAPI) are obtained from Sigma. Alexa Flour 594 or Alexa Flour 488 is from Invitrogen life Technologies. Collagenase/Dispase is from Roche Biosciences, hyaluronidase type V and Dnase I is from Sigma. α-isonitrosopropiophenone for arginase activity and sulphanilamide for nitrite assay is from Sigma. Bouin's solution is from Sigma. MCSF is from Gibco Life technologies and GMCSF from Peprotech Life sciences. D-luciferin potassium salt is from Caliper Life sciences. CD11b magnetic beads are from Militenyi Biotec.RNA isolation kit from Qiagen. Iscript cDNA synthesis kit and SYBR green are from Biorad (Bio-Rad, Hercules, CA).

### *In vivo* tumor experiments

Lewis lung carcinoma (LLC), B16 F10 melanoma and Panc02 cells were obtained from the American Type Culture Collection (ATCC). NB9464D cells were kind gift from Dr. Jon Wigginton. The 9464D disialoganglioside-2-positive, N-myc-overexpressing NB cell line was established in the laboratory of Dr Jon Wigginton (NCI), and was derived from spontaneous NB tumors arising in C57BL/6 N-myc transgenic mice developed originally by Dr William A. Weiss (University of California, San Francisco, CA) [53], [54]. All cells were cultured in DMEM media containing 10% FBS and tested for mycoplasma before implanting in animals. LLC or B16 melanoma cells (1×10^5^) or NB9464 (2×10^6^) were injected subcutaneously into syngeneic 4-6 week old mice. Tumor dimensions were recorded regularly and tumors were harvested 25 days post injection or otherwise stated. Tumor volume was measured using the following formula: Volume = 0.5×length×(width)^2^. Tumors were cryopreserved in O.C.T. or paraffin embedded or collagenase digested for flow cytometric analysis and sorting of macrophages. For experimental metastasis, B16 F10 melanoma cells (5×10^5^) were injected intravenously and lungs were harvested after 15 days. Lungs containing B16 metastases were immersed in Bouin solution to distinguish black tumor colonies from yellowish lung parenchyma. Surface metastatic foci in lung lobes were counted under a dissecting microscope. For spontaneous metastasis, orthotopic pancreatic tumors were initiated by implanting 1×10^6^ Panc02 into the pancreas of syngeneic mice. The abdominal cavities of WT and Rac2-/- were opened and the tails of the pancreata were exteriorized. One million Panc02 cells were injected into the pancreatic tail, the pancreas was placed into the abdominal cavity, and the incision was closed. Pancreatic tumors as well as lymph nodes and other organs with visible metastases were cryopreserved after 30 days of tumor implantation. The metastatic mesentric lymph nodes were counted under a dissecting microscope. All tumor experiments were performed three to four times with n = 8-10. For immunofluoresence studies, cryosections were incubated with primary antibodies against CD31and F4/80 antibodies, followed by Alexa Flour 594 (red) or Alexa488 (green) labeled secondary antibody. The sections were counter stained with DAPI to visualize nuclei and micro vascular density was measured in 40X fields photographed using a Metamorph image capture and analysis software (version 6.3 r5, Molecular Devices). Haematoxylin and eosin staining was performed by the Moores UCSD Cancer Center histology shared resource.

### Tumor digestion and FACS analysis

For FACS analysis, LLC tumors implanted into WT and Rac2-/- mice were excised, minced and digested to single cell population using mixture of enzymes containing 1 mg/ml of collagenase type IV, 10 ug/ml of hyaluronidase type V and 0.01 mg/ml Dnase I for 1 h at 37°C. Cells were solubilized with RBC lysis buffer (Thermo Scientific) and centrifuged. CD11b myeloid cells were purified from tumor cell suspension using the MACS method (Militenyi Biotec) according to the manufacturer's instructions. Briefly, cells were incubated with beads conjugated with anti-mouse CD11b and positively selected on LS columns. The recovered cells were incubated with Fc_γ_R blocking reagent (BD Bioscience) followed by staining with CD11b (clone M1/70, BD Biosciences) and F4/80 (clone BM8, eBioscience) antibodies. To exclude dead cells, 0.5 µg/ml propidium iodide (PI) was added before data acquisition by FACS caliber instrument (BD Bioscience). CD11b and F4/80 positive cells were sorted by FACS and used for real time PCR studies.

### Arginase activity

Arginase activity was measured in cell lysates, as previously described by Coraliza et al. [55]. Briefly, macrophages sorted from LLC tumors injected in WT and Rac2-/- animals were lysed with 100 µl of 0.1% Triton X-100. Subsequently, 100 µl of 50 mM Tris-HCl and 10 µl of 10 mM MnCl2 were added, and the enzyme was activated by heating for 10 min at 56°C. Arginine hydrolysis was conducted by incubating the lysate with 100 µl of 0.5 M L-arginine (pH 9.7) at 37°C for 60 min. The reaction was stopped with 400 µl of H_2_SO_4_ (96%)/H_3_PO_4_ (85%)/H_2_O (1/3/7, v/v/v). The urea concentration was measured at 540 nm after addition of 25 µl of α-isonitrosopropiophenone (dissolved in 100% ethanol), followed by heating at 95°C for 45 min. One unit of enzyme activity is defined as the amount of enzyme that catalyzes the formation of 1 µmol urea per min.

### NOS activity assay

Macrophages sorted from LLC tumors injected in WT and Rac2-/- animals were seeded in triplicates at a concentration of 5×10^5^ per well into 96 well plate and stimulated with 10 ng/ml LPS for the nitric oxide synthesis assay (NOS). After 24 hrs of incubation, equal volumes of culture supernatants (100 µl) were mixed with Greiss reagent (1% sulfanilamide in 5% phosphoric acid and 0.1% *N*-1-naphthylethylenediamine dihydrochloride in double-distilled water). After 10-min incubation at room temperature, the absorbance at 550 nm was measured using microplate plate reader (Bio-Rad). Nitrite concentrations were determined by comparing the absorbance values for the test samples to a standard curve generated by serial dilution of 0.25 mM sodium nitrite.

### Macrophage injection experiments to establish Mθ-autonomous control of tumor growth, metastasis and M1-M2 transition

To determine if the phenotypes observed in the Rac2-/- mice were macrophage autonomous, we reconstituted Rac2-/- mice with bone marrow derived macrophages from wild type mice versus an equal number of BMDM isolated from Rac2-/- animals. LLC tumors (1×10^5^) were subcutaneously implanted into WT and Rac2-/- mice. On 5^th^ day of tumor implantation, 1×10^6^ cultured WT or Rac2-/- BMDM (grown in MCSF) were injected through tail vein in Rac2-/- mice. 1 million WT or Rac2-/- BMDM were injected every third day until tumors were harvested on 25 day. For B16 metastasis reversal experiment, 1 million BMDM were injected two days before B16 (5×10^5^) injections, followed by four macrophage injections (1 million BMDM or GBMDM) every third day for 15 days. Tumors were used for immunofluoresence, immunohistochemistry and for macrophage sorting by FACS as described above. Finally, we performed experiments in which we locally inject Rac2-/- vs WT derived macrophages directly into the lung parenchyma followed by tail vein injection of B16 cells as a metastasis challenge (2 days after injection of Mθs).Rac2-/- mice were intraperitoneally injected with ketamine (60 mg/kg) and xylaxine (20 mg/kg) to induce anesthesia and fixed in either right or left lateral decubitus position after anesthesia. A small cut in the skin near the lungs were made so that lungs are visible. Then 50 µL WT BMDM (1 million) was injected into the upper margin of the sixth intercostal rib on the right anterior axillary line to a depth of about 5 mm rapidly and the needle was promptly pulled out. Similarly, the procedure is performed on the left side, injecting 1 million Rac2-/- BMDMs. The BMDMs and GBMDMs injected in the mice were 90% viable, as determined by trypan blue and replating a small aliquot of cells, at the time of injection. Mice were kept under observation until complete recovery. After two days, 5×10^5^ B16 luciferase cells were injected through tail vein and the luciferase signal was monitored every third day on IVIS (Xenogen IVIS 200) by injecting luciferin intraperitonealy in mice. Mice were sacrificed on day 15 to isolate lungs.

### CD31 staining of neonate retinas

Neonate retinas (P7-8) were isolated from WT, Rac2+/- and Rac2-/- mice and fixed in 4% followed by 2% paraformaldehyde. The retinas were permeabilized with ice cold methanol for 10 min, followed by blocking in 20% normal goat serum. Retinas were incubated with anti-CD31 primary antibodies, followed by incubation with Alexa 488 secondary antibodies. The images were taken under Deltavision deconvolution microscope.

### Isolation of bone marrow-derived macrophages and Rac2 pull down assays

Bone marrow-derived macrophages (BMDM) were isolated as described previously [2]. GBMDMs were cultured on 75 ng/ml of GMCSF. On day 7, most of the adhering cells are macrophages as confirmed by FACS analysis (>90% Mac1 and F4/80 positive cells Rac2 pull down assays were done as described earlier [2]. WT BMDMs were stimulated in 10 cm of non-tissue culture-coated petri dishes coated with 10 µg/ml vitronectin (αvβ_3_), or fragment of fibronectin H296 (α_4_β_1_) or CH271 (α_5_β_1_) or collagen (α_2_β_1_) in PBS for 1 h at 37°C. Cells were scraped, washed in serum-free medium (5×10^6^ cells in 2 ml of serum-free media) and then plated on vitronectin, H296, CH271 or collagen coated plates for 10 min. Following adhesion cells were chilled with Hanks' balanced salt solution at 4°C. For another set of experiments, WT BMDMs or GBMDMs grown in culture for 7 days were serum starved for 4 hrs, followed by stimulation with 50ng/ml of MCSF or GMCSF and cell lysate preparation. Cell lysates were prepared in 25 mM HEPES, pH 7.5, 150 nM NaCl, 1% Igepal CA-630, 10 mM MgCl_2_, 1 mM EDTA, 10% glycerol, 10 µg/ml leupeptin, 10 µg/ml aprotinin, 25 mM sodium fluoride, and 1 mM sodium orthovanadate. Binding reaction was initiated by adding 10 µl of Pak-1-agarose (GST fusion protein, corresponding to the p21 binding CRIB domain, PBD, residues 67–150, of human PAK-1, expressed in *E. coli* and bound to glutathione agarose) to each sample and incubated for 45 min at 4°C and processed as described [8].

### Quantification of gene expression

Total RNA was isolated from BMDMs and sorted tumor macrophages using the Qiagen RNAeasy kit (Qiagen, Hilden, Germany) according to manufacturer's instructions. cDNA was prepared from 1 ug RNA sample using iscript cDNA synthesis kit (Bio-Rad, Hercules, CA). cDNA (2 µL) was amplified by RT-PCR reactions with 1× SYBR green supermix (Bio-Rad, Hercules, CA) in 96-well plates on an CFX96 Real time system (Bio-Rad, Hercules, CA), using the program: 5 min at 95°C, and then 40 cycles of 20 s at 95°C, 1 min at 58°C and 30 sec at 72°C. The primer sets used for different sets of genes are listed in Table S3. Specificity of the produced amplification product was confirmed by examination of dissociation reaction plots. Relative expression levels were normalized to Gapdh expression according to the formula <2∧(Ct gene of interest-Ct Gapdh) > [56]. Values are multiplied by 100 for presentation purposes.

### Microarray analysis

Total RNA was extracted using RNeasy mini columns (Qiagen, Germantown, MD). RNA integrity was assessed using an Agilent 2100 Bio analyzer. All samples demonstrated RNA integrity (RIN) of 7 or greater. RNA was labeled and hybridized to Affymetrix Mouse Genome 1.0 ST arrays. The raw probe intensities were processed using the RMA method to get the gene expression values. 27031 genes expressed in at least four samples were retained. The ComBat method [57] was then used to remove the batch effect. Standard limma analysis [58] was used to identify genes differentially expressed between the Rac2-/-and WT mouse groups (FDR<5%). The microarray data has been assigned an accession number of GSE41236 and can be viewed online on this site http://www.ncbi.nlm.nih.gov/geo/query/acc.cgi?acc=GSE41236.

### Interactome map

Interactome analysis was performed to explore interactions between M1 and M2 driver genes and identify other candidate genes in close network proximity to the known drivers. We first curated M1 and M2 driver lists based on support in literature [19], [33], [59] and our experimental multiple-omic data. Our real time PCR data supports our conclusion that Rac2 controls gene expression of arginase, CLEC10A (Mgl), CD206 (MMR), VEGF, IL1, IL6, TNF, CHI3I3 (YM1), RENTLA (FIZZ1),COX2 and uPA (Figure 4 & 5), hence these genes were used in the preparation of an interactome map. We also obtained a large-scale human protein-protein interaction network from the BioGRID database [60]. We then applied a network propagation algorithm similar to Google's PageRank [61], [62] which seeks to identify novel candidate genes using the “guilt by association” principle, i.e. find candidates among genes which tend to interact densely with the previously identified drivers [63] The interaction network spanning the top 100 genes was identified and annotated with protein and gene expression changes between the Rac2-/- and WT conditions. When new components are identified and validated that regulate M1-M2 transition, they are added to the interactome analysis in an iterative manner.

### Metabolomic profiling

For metabolomic studies, 2–5 million macrophages isolated from WT or Rac2-/- mice and cultured on MCSF (BMDMs) were used. The metabolomic platform consisted of three independent methods: ultrahigh performance liquid chromatography/tandem mass spectrometry (UHLC/MS/MS2) optimized for basic species, UHLC/MS/MS2 optimized for acidic species, and gas chromatography/mass spectrometry (GC/MS). The detailed descriptions of the Metabolon platform, including sample processing, instrument configuration, data acquisition, and metabolite identification and quantitation, were published previously [64], [65]. Essentially, the samples were extracted and split into three equal aliquots for analysis by the three methods. For the two LC methods, chromatographic separation followed by full scan mass spectra was carried out to record retention time, molecular weight (m/z) and MS/MS2 of all detectable ions presented in the samples. For GC, the samples were derivatized using bistrimethyl-silyl-triflouroacetamide. The retention time and molecular weight (m/z) for all detectable ions were measured. The metabolites were identified by comparison of the ion features in the experimental samples to a reference library of chemical standard entries that included retention time, molecular weight (m/z), preferred adducts, and in-source fragments as well as their associated MS/MS2 spectra.

### Cell migration and invasion assay

Integrin-directed cell migration assays (haptotaxis) were performed on polycarbonate membranes using transwell migration chamber (diameter 6.5 mm, pore size 8 µm; Costar Corporation, Cambridge, MA). The underside of the membrane to which cells migrate was coated with 10 µg/ml vitronectin, collagen and fragments of fibronectin H296 (binds with α_4_β_1_) or CH271 (binds with α_5_β_1_) http://www.ncbi.nlm.nih.gov/pubmed/12917394 in PBS for 1 hour at 37°C. Surfaces were subsequently blocked with heat-denatured BSA. Transwells were placed into the lower chamber containing 600 µl serum free media. 2×10^5^ cells (6 days in culture) in 100 µl media/transwell were added to the top of the migration chamber (uncoated side) and allowed to migrate to the coated side of the chamber for 14 h at 37°C. Haptotaxis was quantified as described previously [66]. Invasion assays were performed in triplicate using Transwell invasion chambers coated with Matrigel (BD Biosciences, USA).

### Statistical analysis

Statistical analysis was performed using GraphPad PRISM 4. Where applicable, data were analyzed by unpaired two-tailed *t* test. One or two-way ANOVA with Tukey or Bonferroni post-test, respectively, were used for comparisons of more than two groups.

## Supporting Information

Figure S1 Rac2-/- mice shows no defect in embryonic angiogenesis. Representative photograph showing retinas isolated from WT, Rac2+/− and Rac2-/- neonates (P7-P8). Neovascular endothelial cell are imaged using CD31 immunofluorescent antibody staining (green). Experiment was repeated three times with 4-5 mice in each group.
(TIF)

Figure S2 Rac2 promotes spontaneous metastasis. Representative photograph of the part of colon used in Fig. 2E showing metastatic mesenteric lymph node in WT and not in Rac2-/- and same sections were used for performing H&E staining. Experiment was repeated three times with similar results with 3–4 mice in each group.
(TIF)

Figure S3 Rac2 promotes differentiation of M2 macrophages *in vitro* (A) Heat map generated from microarray analysis of BMDMs isolated from WT and Rac2-/- mice (n = 5 in each group) as described in Materials and Methods. Heatmap shows the differential expression of some novel genes related to macrophage differentiation and function as well as genes related to M1-M2 polarization. (B) Heatmap representation of metabolites across BMDMs from WT (n = 5) and Rac2-/- (n = 5) mice. Shades of yellow represent elevation of a metabolite and shades of blue represent decrease of a metabolite relative to the median metabolite levels (see color scale). Fig. shows the higher expression of metabolites related to nucleotide metabolism in Rac2-/- BMDMs.
(TIF)

Figure S4 Phenotype reversal by WT BMDMs and not by GBMDMs or Rac2-/- BMDMs (A) Quantitative PCR analysis of mRNA for M1, M2 specific genes in the BMDMs or GBMDMs cultured in MCSF or GMCSF respectively. (B) Quantitative PCR analysis of mRNA for IL 1, IL 6, TNFα, cox2, uPA, MMP9, MMR, Arginase and TGF-β in the macrophages sorted from LLC tumors implanted in Rac2-/- mice and treated with WT BMDMs or GBMDMs as described in Fig. 6B & C subpanel. Values are mean ± SEM (n = 3–4). Statistical significance is assessed by two sample *t*-test where *denotes *P*<0.05, ** denotes *P*<0.01 and *** denotes *P*<0.001. Experiment was repeated three times with similar results. (C) Figure shows the mean number of tumor nodules visible on the surface of the lungs in Rac2-/- mice treated with 1 million WT BMDMs or WT GBMDMs. Values are mean ± SEM (*n* = 5–6-; *P*<0.001; pair wise two-sided Student's *t* test). (D) H &E staining sections showing pulmonary metastasis in Rac2-/- mice injected with local injection of 1 million WT BMDM in the right lobe of lung and WT GBMDM or Rac2-/- BMDM in the left lobe of Rac2-/- mice, followed by tail vein injections of 5×10^5^ B16 luciferase cells.
(TIF)

Table S1 List of genes expressed differentially in microarray analysis in WT versus Rac2-/- BMDM. Fold change represents the gene expression value in WT BMDMs divided by gene expression values of same gene in Rac2-/- BMDMs; If gene expression is increased in WT BMDMs the fold-change is preceded by an up arrow (↑), if gene expression in WT is decreased relative to Rac2-/-, the fold-change value is preceded by a down arrow (↓). Gene functions are based on literature, with emphasis placed on functions in the macrophage whenever possible.
(DOC)

Table S2 List of top 100 genes in an interactome map, identified as novel candidate genes/proteins by multiple-omic analysis which tend to interact densely with the previously identified drivers of M1-M2 transition. This represents an iterative process; when new components are validated they are added to the interactome query.
(DOC)

Table S3 List of primers used in real time PCR.
(DOC)
