# Supplementary figures and images for: Rac2 Controls Tumor Growth, Metastasis and M1-M2 Macrophage Differentiation In Vivo
Source: PLoS One. 2014 Apr 25;9(4):e95893. doi: 10.1371/journal.pone.0095893 (PMC4000195; doi:10.1371/journal.pone.0095893)

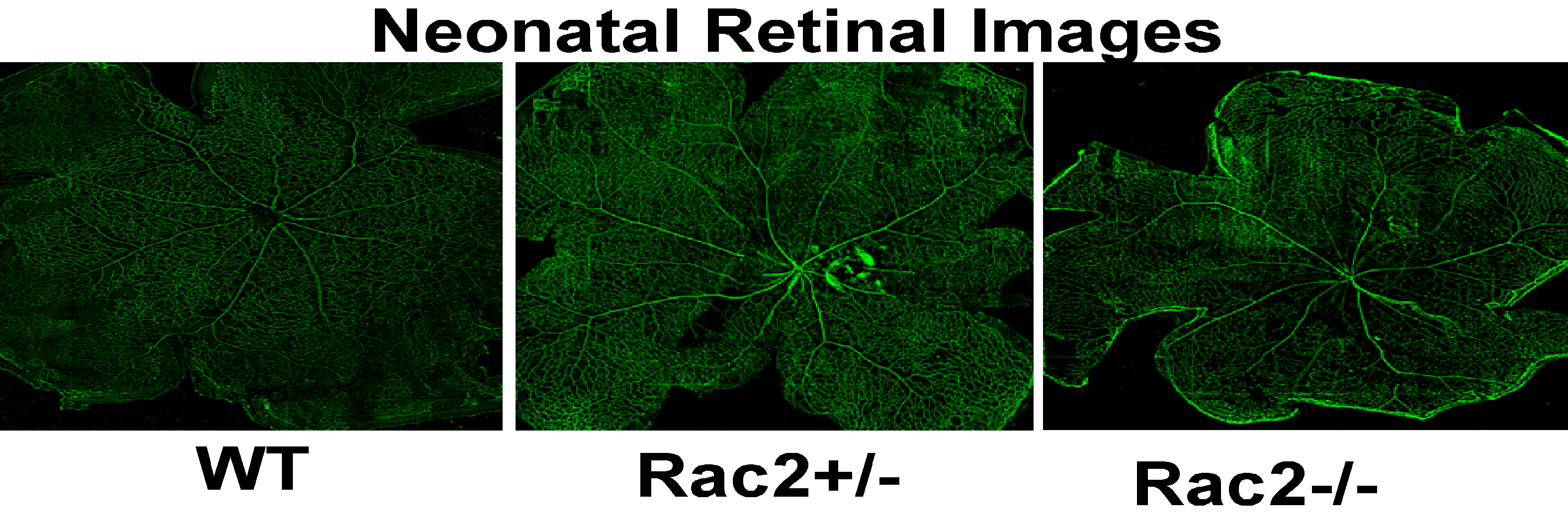

Supplement: Figure S1 — Rac2-/- mice shows no defect in embryonic angiogenesis. Representative photograph showing retinas isolated from WT, Rac2+/− and Rac2-/- neonates (P7-P8). Neovascular endothelial cell are imaged using CD31 immunofluorescent antibody staining (green). Experiment was repeated three times with 4-5 mice in each group. (TIF) [file pone.0095893.s001.tif]

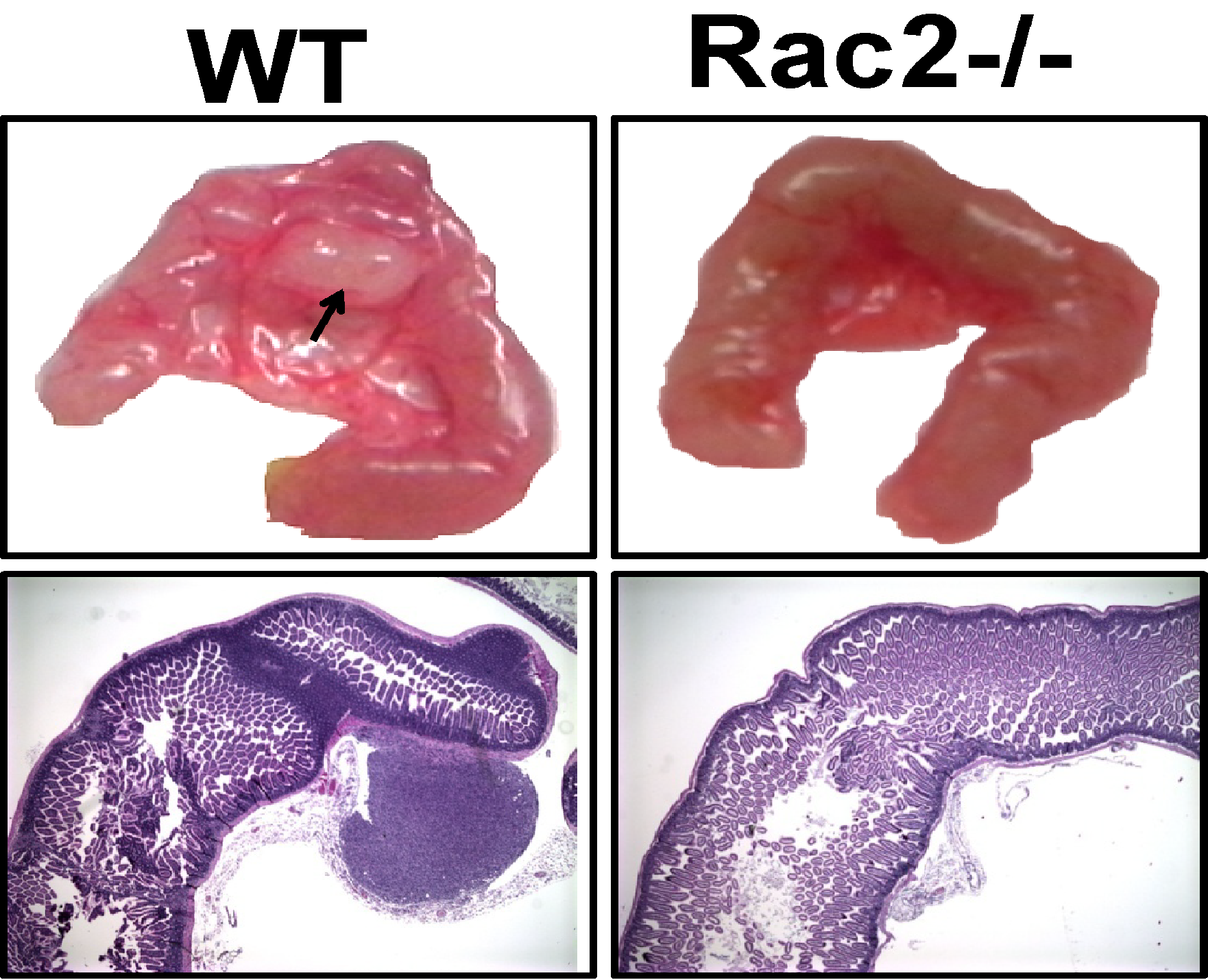

Supplement: Figure S2 — Rac2 promotes spontaneous metastasis. Representative photograph of the part of colon used in Fig. 2E showing metastatic mesenteric lymph node in WT and not in Rac2-/- and same sections were used for performing H&E staining. Experiment was repeated three times with similar results with 3–4 mice in each group. (TIF) [file pone.0095893.s002.tif]

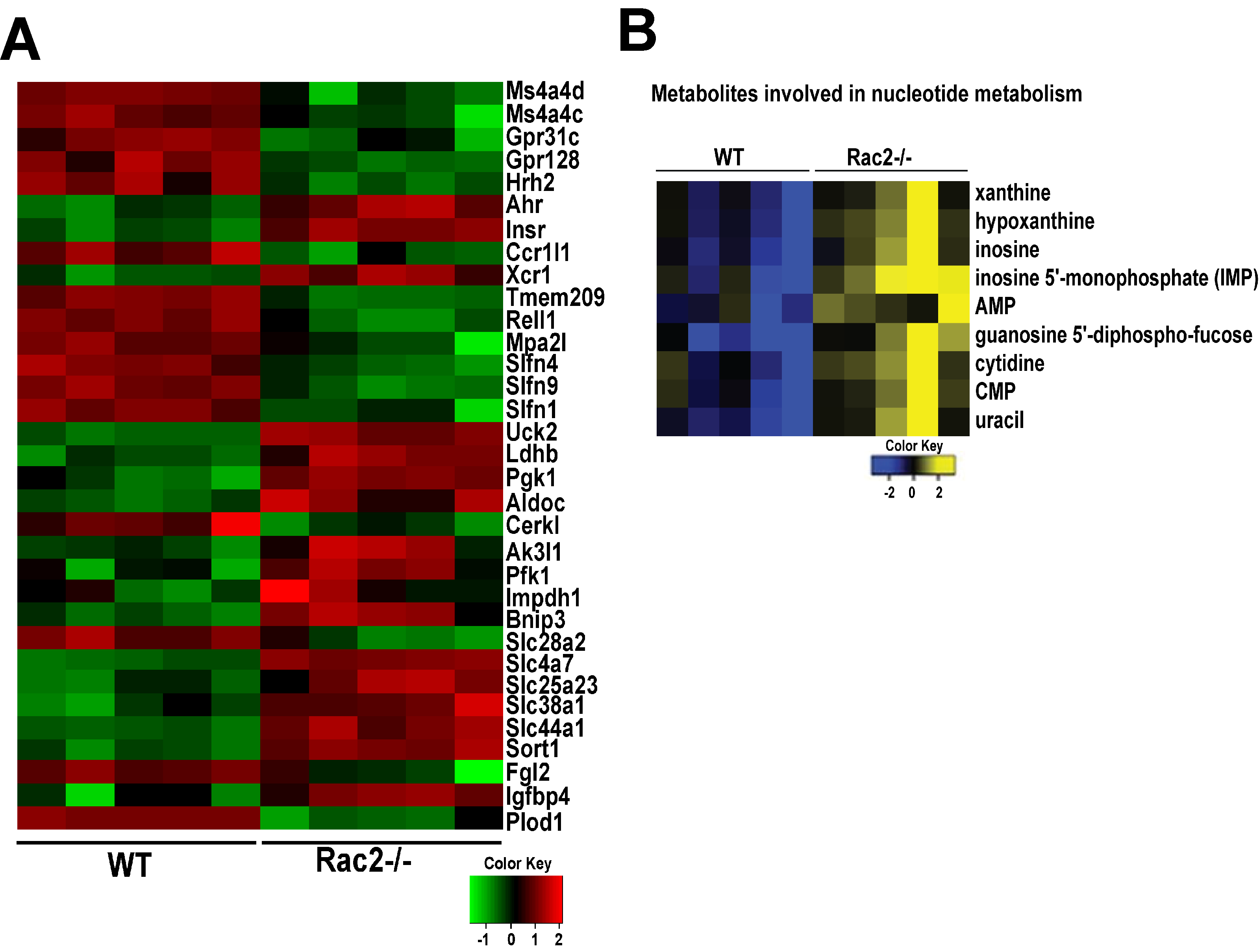

Supplement: Figure S3 — Rac2 promotes differentiation of M2 macrophages in vitro (A) Heat map generated from microarray analysis of BMDMs isolated from WT and Rac2-/- mice (n = 5 in each group) as described in Materials and Methods. Heatmap shows the differential expression of some novel genes related to macrophage differentiation and function as well as genes related to M1-M2 polarization. (B) Heatmap representation of metabolites across BMDMs from WT (n = 5) and Rac2-/- (n = 5) mice. Shades of yellow represent elevation of a metabolite and shades of blue represent decrease of a metabolite relative to the median metabolite levels (see color scale). Fig. shows the higher expression of metabolites related to nucleotide metabolism in Rac2-/- BMDMs. (TIF) [file pone.0095893.s003.tif]

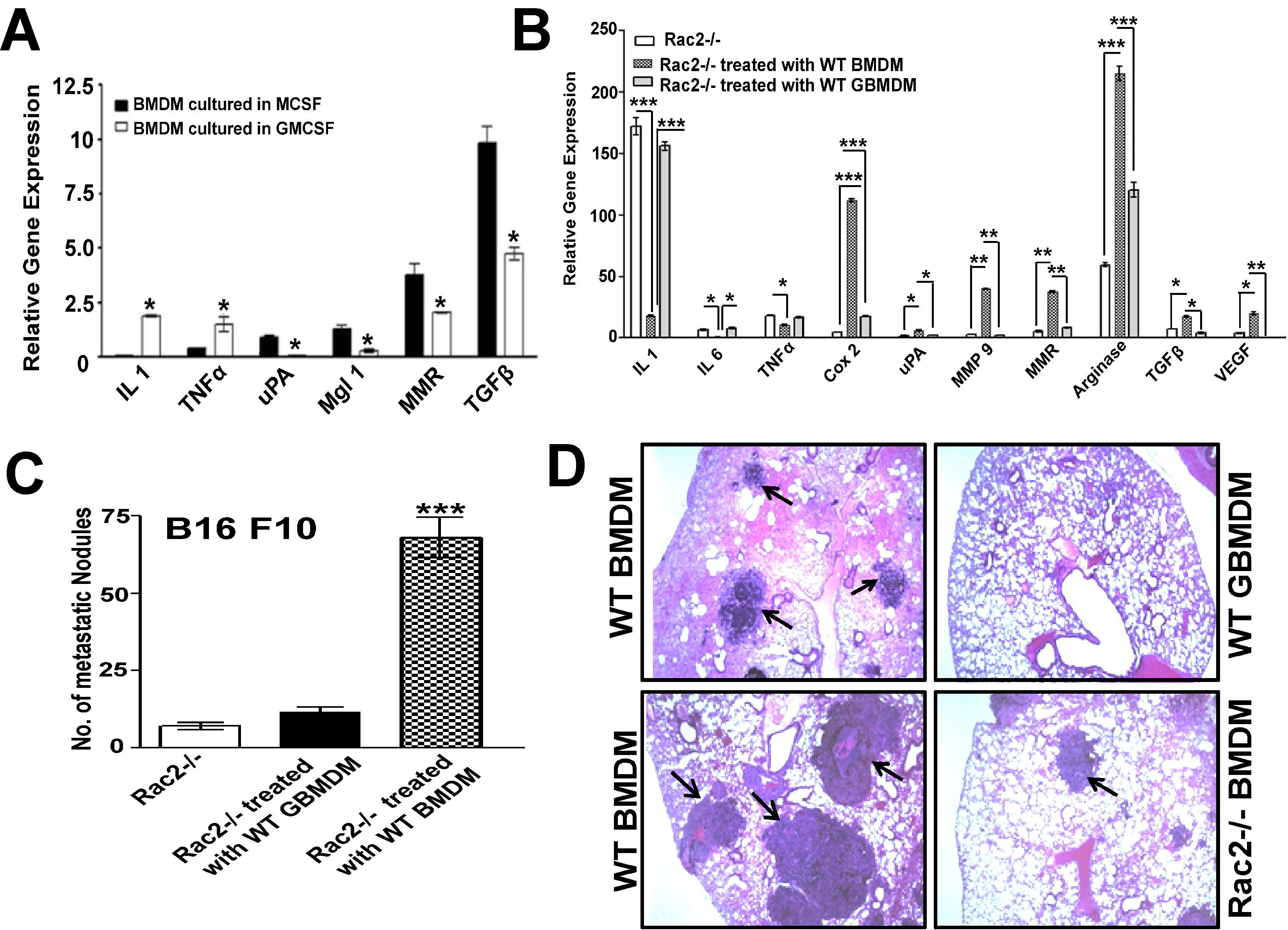

Supplement: Figure S4 — Phenotype reversal by WT BMDMs and not by GBMDMs or Rac2-/- BMDMs (A) Quantitative PCR analysis of mRNA for M1, M2 specific genes in the BMDMs or GBMDMs cultured in MCSF or GMCSF respectively. (B) Quantitative PCR analysis of mRNA for IL 1, IL 6, TNFα, cox2, uPA, MMP9, MMR, Arginase and TGF-β in the macrophages sorted from LLC tumors implanted in Rac2-/- mice and treated with WT BMDMs or GBMDMs as described in Fig. 6B & C subpanel. Values are mean ± SEM (n = 3–4). Statistical significance is assessed by two sample t-test where *denotes P<0.05, ** denotes P<0.01 and *** denotes P<0.001. Experiment was repeated three times with similar results. (C) Figure shows the mean number of tumor nodules visible on the surface of the lungs in Rac2-/- mice treated with 1 million WT BMDMs or WT GBMDMs. Values are mean ± SEM (n = 5–6-; P<0.001; pair wise two-sided Student's t test). (D) H &E staining sections showing pulmonary metastasis in Rac2-/- mice injected with local injection of 1 million WT BMDM in the right lobe of lung and WT GBMDM or Rac2-/- BMDM in the left lobe of Rac2-/- mice, followed by tail vein injections of 5×10^5^ B16 luciferase cells. (TIF) [file pone.0095893.s004.tif]
