# Supplementary material for: Rac2 Controls Tumor Growth, Metastasis and M1-M2 Macrophage Differentiation In Vivo
Source: PLoS One. 2014 Apr 25;9(4):e95893. doi: 10.1371/journal.pone.0095893 (PMC4000195; doi:10.1371/journal.pone.0095893)
Supplement: Table S1 — List of genes expressed differentially in microarray analysis in WT versus Rac2-/- BMDM. Fold change represents the gene expression value in WT BMDMs divided by gene expression values of same gene in Rac2-/- BMDMs; If gene expression is increased in WT BMDMs the fold-change is preceded by an up arrow (↑), if gene expression in WT is decreased relative to Rac2-/-, the fold-change value is preceded by a down arrow (↓). Gene functions are based on literature, with emphasis placed on functions in the macrophage whenever possible. (DOC) [file pone.0095893.s005.doc]

**Table S1**.

| **Pathway/Function** | **Gene** | **Symbol** | **Fold Change** | **Function** |
| --- | --- | --- | --- | --- |
| **Cytokines and Chemokines**  **VEGF signaling Pathway**  **Invasion and Angiogeneis**  **Cell Cycle**  **Membrane Receptors**  **Enzymes**  **Apoptosis related protein**  **Solute Carrier**  **Extracellular mediators** | Chemokine(C-C) motif ligand 8  Chemokine(C-C) motif ligand 22  Chemokine(C-C) motif ligand 2  Chemokine(C-C) motif ligand 7  Chemokine(C-C) motif ligand 12  Interferon alpha inducible protein 27 like 1  Tumor Necrosis Factor alpha induced protein 8  Kinase insert domain repeat  FMS-related tyrosine Kinase 1  Matrix metallopeptidase 9  Matrix metallopeptidase 25  Matrix metallopeptidase 13  Cyclin D1  Cyclin D2  Membrane-spanning- 4- domains Subfamily A, member 4D  Membrane spanning 4 domains Subfamily A, member 4C  G protein-coupled receptor 31  G protein-coupled receptor 128  Histamine Receptor H2  Aryl -hydrocarbon Receptor  Insulin Receptor  Chemokine (C-C) motif receptor 1 like 1  Chemokine (C motif) receptor 1  Transmembrane protein 209  RELT like 1  Macrophage activation 2 like  Schlafen4  Schlafen9  Schlafen1  Uridine-cytidine kinase2  Lactate dehydrogenase B  Phosphoglycerate kinase1  Aldolase C  Ceramide kianse like  Adenylate kinase 3 like 1  Phosphofructokinase1  Inosine monophosphate dehydrogenase  BCL2 adenovirus E1B interacting protein 3  Solute Carrier family protein family 28, member 2  Solute Carrier family protein family 4, member 7  Solute Carrier family protein family 25, member 23  Solute Carrier family protein family 38, member1  Solute Carrier family protein family 44, member 23  Sortilin1  Fibrinogen like 2  Insulin like growth factor binding protein4  Procollagen lysine hydroxylase | Ccl8  Ccl22  Ccl2  Ccl7  Ccl12  Ifi27l1  Tnfaip8  Kdr  Flt1, VEGFR1  MMP9  MMP25  MMP13  Ccnd 1  Ccnd 2  Ms4a4d  Ms4a4c  Gpr31c  Gpr128  Hrh2  Ahr  Insr  Ccr1l1  Xcr1  Tmem209  Rell1  Mpa2l  Slfn4  Slfn9  Slfn1  Uck2  Ldhb  Pgk1  Aldoc  Cerkl  Ak3l1  Pfk1  Impdh1  Bnip3  Slc28a2  Slc4a7  Slc25a23  Slc38a1  Slc44a1  Sort1  Fgl2  Igfbp4  Plod1 | 1.2↑  1.08↑  1.15↑  1.19↑  1.2↑  0.86↓  0.96↓  1.13↑  1.15↑  1.19↑  1.1↑  1.13↑  1.14↑  0.97↑  1.18↑  1.08↑  1.13↑  1.25↑  1.04↑  0.86↓  0.93↓  1.15↑  0.89↓  1.08↑  1.04↑  1.18↑  1.47↑  1.15↑  1.29↑  0.79↓  0.84↓  0.96↓  0.88↓  1.08↑  0.84↓  0.92↓  0.95↓  0.92↓  1.16↑  0.866↓  0.877↓  0.97↓  0.95↓  0.80↓  1.13↑  0.92↓  1.07↑ | Chemotactic for and activates monocytes, T cells and mast cells that are involved in inflammatory response  M2 macrophage marker. Specifically attracts monocyte and regulate macrophage function.  M2 macrophage marker.  Recruits monocyte, memory T cells and dendritic cells to sites of infection and inflammation  Specifically attracts monocytes and regulate macrophage function  Specifically attracts monocytes, eosinophills and lymphocytes  Promotes cell death. Mediates IFN-induced apoptosis characterized by a rapid and robust release of cytochrome C from the mitochondria and activation of BAX and caspases 2, 3, 6, 8 and 9  Suppressor of TNF alpha induced apoptosis  Main mediator for VEGF-induced endothelial proliferation, survival, migration, tubular morphogenesis and sprouting.  Angiogenesis, patterning of blood vessels, cell adhesion, cell differentiation, cell migration.  Breakdown of extracellular matrix proteins, tumor associated tissue remodeling, role in tumor growth and metastasis  Tumor invasion and metastasis through activation of MMP2  Cell migration, angiogenesis, tumor growth and host defence  Macrophage adhesion, motility and migration  Mediator of cell division,  Macrophage activation by LPS  This gene encodes a B-lymphocyte surface molecule which plays a role in the development and differentiation of B-cells into plasma cells.  unknown  Shows high affinity for the lipid mediator 12-lipoxygenase-derived product 12-(S)-hydroxy-5,8,10,14-eicosatetraenoic acid (HETE  Member of adhesion GPCR family of receptors  Inflammatory role in macropahges.  ligand-activated transcription factor involved in immunoregulatory role in macrophages and T cells  involved in expression of eNOS in endothelial cells  involved in C-C chemokine activity and G-protein coupled receptor activity  also known as GPR5. involved in inflammatory response.  unknown  unknown  unknown  Involved in macrophage activation and differentiation  unknown  Promotes cell growth and development of T lymphocytes  Involved in nucleotide metabolism pathway  Converts pyruvate final product of glycolysis to lactate  Major enzyme used in glycolysis in the first ATP generating step of glycolytic pathway.  Enzyme of glycolysis and converts fructose1, 6 bisphosphate into glyceraldehydes 3 phosphate and dihydroxyacetone phosphate.  Play important role in cell proliferation and survival  Involved in nucleotide metabolism pathway and catalyzes the interconversion of adenine nucleotides  Enzyme of glycolysis, phosphorylates fructose-6-phosphate  Enzyme involved in nucleotide metabolism converts inosine monophosphate to xanthine monophosphate  Known to induce apoptosis  Involved in homeostasis of endogenous nucleosides  Mediates transport of sodium and bicarbonate ions  Gene functions as a gated pore that translocates ADP from mitochondrial matrix to cytoplasm  Important transporter of glutamine an intermediate in the detoxification of ammonia  Amino acid transporters play essential role in uptake of nutrients, production of energy  Participates in degradation of VLDL  Found on surface of macrophages and involved in immune and adaptive response  Serve as carrier protein for IGF1  Attach hydroxyl group to lysine in collagen like proteins |
