# Supplementary material for: Rac2 Controls Tumor Growth, Metastasis and M1-M2 Macrophage Differentiation In Vivo
Source: PLoS One. 2014 Apr 25;9(4):e95893. doi: 10.1371/journal.pone.0095893 (PMC4000195; doi:10.1371/journal.pone.0095893)
Supplement: Table S2 — List of top 100 genes in an interactome map, identified as novel candidate genes/proteins by multiple-omic analysis which tend to interact densely with the previously identified drivers of M1-M2 transition. This represents an iterative process; when new components are validated they are added to the interactome query. (DOC) [file pone.0095893.s006.doc]

**Table S2.**

| **Gene Symbol** | **Gene name** |
| --- | --- |
| HNRNPD | Heterogeneous nuclear ribonucleoprotein D |
| CUL1 | Cullin 1 |
| HRSP12 | Heat-Responsive Protein 12 |
| COX2 | Cyclooxygenase 2 |
| VEGFA | Vascular Endothelial Growth Factor A |
| UBC | Ubiquitin C |
| HSP90AA1 | [Heat Shock Protein 90kDa Alpha (Cytosolic), Class A Member 1](http://www.genenames.org/data/hgnc_data.php?hgnc_id=5253) |
| MDM2 | Mouse double minute 2 homolog |
| SNCA | Synuclein, Alpha |
| CRYAB | Crystallin, alpha B |
| HIF1A | Hypoxia inducible factor 1, alpha subunit |
| CDK2 | Cyclin-dependent kinase 2 |
| HSPA4 | Heat shock 70kDa protein 4 |
| NOS2 | Nitric oxide synthase 2, inducible |
| ESR1 | Estrogen receptor 1 |
| IL1A | Interleukin-1 Alpha |
| S100A13 | S100 calcium binding protein A13 |
| DLD | Dihydrolipoamide dehydrogenase |
| NHLRC2 | NHL repeat containing 2 |
| TNFAIP8 | Tumor necrosis factor, alpha-induced protein 8 |
| ASS1 | Argininosuccinate synthase 1 |
| CCL2 | Chemokine (C-C motif) ligand 2 |
| DARC | Duffy blood group, atypical chemokine receptor |
| EIF6 | [Eukaryotic Translation Initiation Factor 6](http://www.genenames.org/data/hgnc_data.php?hgnc_id=6159) |
| HAX1 | HCLS1 associated protein X-1 |
| VCAM1 | Vascular cell adhesion molecule 1 |
| ATG3 | Autophagy related 3 |
| ICT1 | Immature colon carcinoma transcript 1 |
| HSPA9 | Heat shock 70kDa protein 9 |
| FN1 | Fibronectin 1 |
| SHC1 | Src homology 2 domain containing |
| CLEC10A | C-type lectin domain family 10, member A, |
| IL6 | Interleukin 6 |
| IL6R | Interleukin 6 receptor |
| CHI3L3 | Chitinase 3-like 3 |
| CD206 | Mannose receptor, C type 1 |
| CCR3 | Chemokine (C-C motif) receptor 3 |
| CCR4 | Chemokine (C-C motif) receptor 4 |
| CCL5 | Chemokine (C-C motif) ligand 5 |
| Ccr2 | Chemokine (C-C motif) receptor 2 |
| CCL22 | Chemokine (C-C motif) ligand 22 |
| DPP4 | Dipeptidyl-peptidase 4 |
| VTN | Vitronectin |
| THBS2 | Thrombospondin 2 |
| PGF | Placental growth factor |
| VEGFB | Vascular Endothelial Growth Factor B |
| ADA2 | Adenosine deaminase 2 |
| Nedd4 | Neural precursor cell expressed, developmentally down-regulated 4 |
| AHC1 | Subunit of the Ada histone acetyltransferase complex |
| GCN5 | General Control Of Amino-Acid Synthesis 5-Like 2 (Yeast) |
| NGG1 | Novel yeast gene required for glucose repression of GAL4p-regulated transcription |
| HFI1 | Hypoxia inducible factor 1 |
| SERPINE1 | Serine proteinase inhibitor (serpin) superfamily |
| MED4 | Mediator complex subunit 4 |
| NDN | Necdin |
| TGFB1 | Transforming growth factor, beta 1 |
| ORC2 | Origin recognition complex, subunit 2 |
| ORC4 | Origin recognition complex, subunit 4 |
| THBS1 | Thrombospondin 1 |
| GCLC | Glutamate-cysteine ligase, catalytic subunit |
| ALDH4A1 | Aldehyde dehydrogenase 4 family, member A1 |
| UCHL5 | Ubiquitin carboxyl- terminal hydrolase L5 |
| HERC3 | HERC ubiquitin ligase 3 |
| SH3GL2 | SH3-domain GRB2-like 2 |
| SPT7 | Suppressors of retrotransposon insertion mutations member 7 |
| CDC45 | Cell division cycle 45 |
| USP53 | Ubiquitin specific peptidase 53 |
| VCAN | Versican |
| CDC6 | Cell division cycle 6 |
| PCNA | Proliferating cell nuclear antigen |
| PLOD1 | Procollagen-lysine 1, 2-oxoglutarate 5- dioxygenase 1 |
| PLCG1 | Phospholipase C, gamma 1 |
| FLT1 | Fms-related tyrosine kinase 1 |
| SHC2 | Src homology 2 domain containing |
| NEDD4 | Neural precursor cell expressed, developmentally down-regulated 4 |
| C12orf44 | Chromosome 12 open reading frame 44 |
| ARG1 | Arginase 1 |
| CXCL5 | Chemokine (C-X-C motif) ligand 5 |
| MMP9 | Matrix metallopeptidase 9 |
| MCM3 | Minichromosome maintenance complex component 3 |
| MCM2 | Minichromosome maintenance complex component 2 |
| IPO5 | Importin 5 |
| CXCL6 | Chemokine (C-X-C motif) ligand 6 |
| SPT8 | Suppressors of retrotransposon insertion mutations, member 8 |
| DPP3 | Dipeptidyl-peptidase 3 |
| MYC | Retroviral myconcogene |
| TNF | Tumor necrosis factor |
| CAV1 | Caveolin 1 |
| ACOT7 | Acyl-CoA thioesterase 7 |
| KDR | Kinase insert domain receptor |
| ELAVL1 | Embryonic lethal, abnormal vision, Drosophila)-like 1 |
| PPP1R2 | Protein phosphatase 1, regulatory (inhibitor) subunit 2 |
| PTP4A1 | Protein tyrosine phosphatase type IVA, member 1, |
| YWHAE | Tyrosine 3- monooxygenase/tryptophan 5-monooxygenase activation protein |
| BTRC | Beta-transducin repeat containing E3 ubiquitin protein ligase |
| PTP4A2 | Protein tyrosine phosphatase type IVA, member 2 |
| PSMA1 | Proteasome (Prosome, Macropain) Subunit, Alpha Type, 1 |
| MCM7 | Minichromosome maintenance complex component 7 |
| RAD21 | Double-strand-break repair protein rad21 homolog |
| PLAU | Plasminogen activator, urokinase |
