# Supplementary material for: Rac2 Controls Tumor Growth, Metastasis and M1-M2 Macrophage Differentiation In Vivo
Source: PLoS One. 2014 Apr 25;9(4):e95893. doi: 10.1371/journal.pone.0095893 (PMC4000195; doi:10.1371/journal.pone.0095893)
Supplement: Table S3 — List of primers used in real time PCR. (DOC) [file pone.0095893.s007.doc]

**Table S3**

| **Gene** | **Forward Primer (5’-3’)** | **Reverse Primer (5’-3’)** |
| --- | --- | --- |
| **IL 1β** | CAACCAACAAGTGATATTCTCCATG | GATCCACACTCTCCAGCTGCA |
| **IL 6** | GAGGATACCACTCCCAACAGACC | AAGTGCATCATCGTTGTTCATACA |
| **IL 10** | GGT TGC CAA GCC TTA TCG GA | ACC TGC TCC ACT GCC TTG CT |
| **IL 23** | TGTGCCCCGTATCCAGTGT | CGGATCCTTTGCAAGCAGAA |
| **TNF α** | CATCTTCTCAAAATTCGAGTGACAA | TGGGAGTAGACAAGGTACAACCC |
| **TGF β** | TGACGTCACTGGAGTTGTACGG | GGTTCATGTCATGGATGGTGC |
| **uPA** | TTA CTG CAG GAA CCC TGA CAA CCA | TGC TAA GAG AGC AGT CAT GCA CCA |
| **Cox 2** | GTATCAGAACCGCATTGCCTC | CGGCTTCCAGTATTGAGGAGAACAGAT |
| **MMP 2** | CAG GGA ATG AGT ACT GGG TCT ATT | ACT CCA GTT AAA GGC AGC ATC TAC |
| **MMP 9** | AAT CTC TTC TAG AGA CTG GGA AGG AG | AGC TGA TTG ACT AAA GTA GCT GGA |
| **VEGF** | ATC CGC AGA CGT GTA AAT GTT CCT | TCA CCG CCT TGG CTT GTC AC |
| **MGL 1** | ATG ATG TCT GCC AGA GAA CC | ATC ACA GAT TTC AGC AAC CTT A |
| **MMR** | GCA AAT GGA GCC GTC TGT GC | CTC GTG GAT CTC CGT GAC AC |
| **YM 1** | GGG CAT ACC TTT ATC CTG AG | CCA CTG AAG TCA TCC ATG TC |
| **Arginase** | ATG GAA GAG ACC TTC AGC TAC | GCT GTC TTC CCA AGA GTT GGG |
| **GAPDH** | ACC ACA GTC CAT GCC ATC AC | TCC ACC ACC CTG TTG CTG TA |
